# Structure, dynamics, coding and optimal biophysical parameters of efficient excitatory-inhibitory spiking networks

**DOI:** 10.1101/2024.04.24.590955

**Published:** 2024-04-27

**Authors:** Veronika Koren, Simone Blanco Malerba, Tilo Schwalger, Stefano Panzeri

**Affiliations:** 1 Institute of Neural Information Processing, Center for Molecular Neurobiology (ZMNH), University Medical Center Hamburg-Eppendorf (UKE), 20251 Hamburg, Germany; 2 Institute of Mathematics, Technische Universität Berlin, 10623 Berlin, Germany; 3 Bernstein Center for Computational Neuroscience Berlin, 10115 Berlin, Germany

**Keywords:** Neural coding, Efficient coding, Spiking neural networks, Recurrent Neural Networks, Population coding, Spike-triggered adaptation, Integrate-and-fire neuron, Excitatory-Inhibitory balance, Connectivity, Optimality

## Abstract

The principle of efficient coding posits that sensory cortical networks are designed to encode maximal sensory information with minimal metabolic cost. Despite the major influence of efficient coding in neuroscience, it has remained unclear whether fundamental empirical properties of neural network activity can be explained solely based on this normative principle. Here, we rigorously derive the structural, coding, biophysical and dynamical properties of excitatory-inhibitory recurrent networks of spiking neurons that emerge directly from imposing that the network minimizes an instantaneous loss function and a time-averaged performance measure enacting efficient coding. The optimal network has biologically-plausible biophysical features, including realistic integrate-and-fire spiking dynamics, spike-triggered adaptation, and a non-stimulus-specific excitatory external input regulating metabolic cost. The efficient network has excitatory-inhibitory recurrent connectivity between neurons with similar stimulus tuning implementing feature-specific competition, similar to that recently found in visual cortex. Networks with unstructured connectivity cannot reach comparable levels of coding efficiency. The optimal biophysical parameters include 4 to 1 ratio of excitatory vs inhibitory neurons and 3 to 1 ratio of mean inhibitory-to-inhibitory vs. excitatory-to-inhibitory connectivity that closely match those of cortical sensory networks. The efficient network has biologically-plausible spiking dynamics, with a tight instantaneous E-I balance that makes them capable to achieve efficient coding of external stimuli varying over multiple time scales. Together, these results explain how efficient coding may be implemented in cortical networks and suggests that key properties of biological neural networks may be accounted for by efficient coding.

## Introduction

Information about the sensory world is represented in the brain through the dynamics of neural population activity^1,2^. One prominent theory about the principles that may guide the design of neural computations for sensory function is efficient coding^3,4,5^. This theory posits that neural computations are optimized to maximize the information that neural systems encode about features of sensory stimuli while at the same time limiting the metabolic cost. Efficient coding has been highly influential, especially in visual neuroscience and computational vision^6,7,8,9^, and has been developed to become a normative theory of how networks are organized and designed to optimally process natural sensory stimuli in visual^10,11^, auditory^12^ and olfactory sensory pathways^13^.

The first normative neural network models^4,11^ designed with efficient coding principles had at least two major levels of abstractions. First, information was assumed to be processed in a purely feedforward manner, whereas information processing in real neural circuits often involves recurrent or feedback computations. Second, neural dynamics was greatly simplified, ignoring the spiking nature of neural activity. Instead, in biological networks considerable amount of information are encoded or transmitted only through the millisecond-precise timing of spikes^14,15,16,17,18,19,20^. Also, these earlier works mostly considered encoding of static sensory stimuli, whereas the sensory environment changes continuously at multiple timescales and the dynamics of neural networks encodes these temporal variations of the environment^21,22,23,24^.

Recent years have witnessed a considerable effort and success in laying down the mathematical tools and methodology to understand how to formulate efficient coding theories of neural networks with much more biological realism^25^. This work has established the incorporation of recurrent connectivity^26,27^, of spiking neurons, and of time-varying stimulus inputs^28,29,30,31,32,33,34,35^. In these models, the efficient coding principle has been implemented by designing networks whose activity minimizes the encoding accuracy (the error between a desired representation and a linear readout of network’s activity) subject to a constraint on the metabolic cost of processing (proportional to the total number of spikes fired by a population of neurons). This double objective is captured by a loss function that trades-off encoding accuracy and metabolic cost. The minimization of the loss function is performed through a greedy approach, by assuming that a neuron will emit a spike only if this will decrease the loss. This, in turn, yields a set of leaky integrate-and-fire (LIF) neural equations which govern the network dynamics^28^, which can also include biologically plausible non-instantaneous synaptic delays^36,35,34^. These previous implementations, however, had neurons that did not respect Dale’s law. In recent work^37^, we further extended the biological plausibility of these models by analytically deriving how to implement efficient coding in networks of spiking neurons that respect Dale’s law. These networks take the form of generalized leaky integrate-and-fire (gLIF) models of excitatory (E) and inhibitory (I) neurons endowed with spike-triggered adaptation^38,39,40^, which can provide highly accurate predictions of spike times in biological networks^41^. Efficient spiking models have thus the potential to provide a unifying theory of neural coding through spiking dynamics of E-I circuits^42,37^ with elements that are fully biologically plausible and potentially interpretable as biophysical variables.

However, despite the major progress described above, as well the progress provided by other studies of efficient coding with spikes^31,43,44,33,29^, we still lack a thorough characterization of which structural, coding, biophysical and dynamical properties of excitatory-inhibitory recurrent networks of spiking neurons are directly related to efficient coding principles. Previous studies only rarely made predictions that could be quantitatively compared against experimentally measurable properties of biological neural networks. As a consequence, we still do not know which, if any, fundamental properties of cortical networks emerge directly from imposing efficient coding.

To address the above questions, we analyze systematically our biologically plausible efficient coding model of E and I neurons that respect Dale’s law^37^ to make concrete predictions about experimentally measurable structural, coding and dynamical features of neural activity that arise from efficient coding. We systematically investigated how experimentally measurable emergent dynamical properties, such as firing rates, trial-to-trial spiking variability of single neurons, E-I balance^45^ and noise correlations, relate to optimally-efficient coding. We further analyze how the organization of the connectivity arising by imposing efficient coding relates to the anatomical and effective connectivity recently reported in visual cortex, which suggests competition between excitatory neurons with similar stimulus tuning. We found that several key and robustly found empirical properties of cortical circuits match the predictions of our efficient coding network, lending support to the notion that efficient coding may be a design principle that has shaped the evolution of cortical circuits and that may be used to conceptually understand and interpret them.

## Results

### Assumptions and emergent properties of the efficient E-I network derived from first principles

We study the properties of a spiking neural network in which the dynamics and structure of the network are analytically derived starting from first principles of efficient coding of sensory stimuli. The model relies on a number of assumptions, described next.

**Figure 1.**
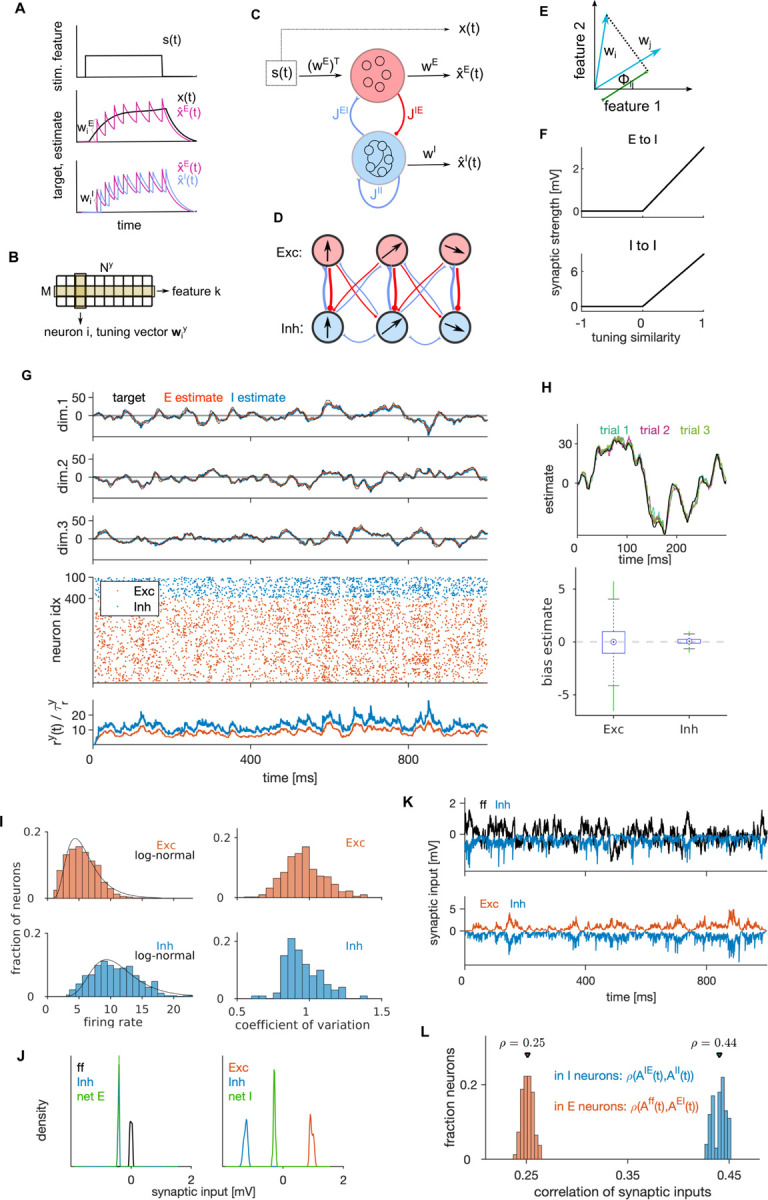
Structural and dynamical properties of the efficient E-I spiking network. **(A)** Encoding of a target signal representing the evolution of a stimulus feature (top) with one E (middle) and one I spiking neuron (bottom). The target signal $x\left(t\right)$ integrates the input signal $s\left(t\right)$. The readout of the E neuron tracks the target signal and the readout of the I neuron tracks the readout of the E neuron. Neurons spike to bring the readout of their activity closer to their respective target. Each spike causes a jump of the readout, with the sign and the amplitude of the jump being determined by neuron’s tuning parameters. **(B)** Schematic of the matrix of tuning parameters. Every neuron is selective to all stimulus features (columns of the matrix), and all neurons participate in encoding of every feature (rows). **(C)** Schematic of the network with E (red) and I (blue) cell type. E neurons are driven by the stimulus features while I neurons are driven by the activity of E neurons. E and I neurons are connected through recurrent connectivity matrices. **(D)** Schematic of E (red) and I (blue) synaptic interactions. Arrows represent the direction of the tuning vector of each neuron. Only neurons with similar tuning are connected. **(E)** Schematic of similarity of tuning vectors (tuning similarity) in a 2-dimensional space of stimulus features. **(F)** Synaptic strength as a function of tuning similarity. **(G)** Coding and dynamics in a simulation trial. Top three rows show the signal (black), the E estimate (red) and the I estimate (blue) in each of the three stimulus dimensions. Below are the spike trains. In the bottom row, we show the average instantaneous firing rate (in Hz). **(H)** Top: Example of the target signal (black) and the E estimate in 3 simulation trials (colors) in one signal dimension. Bottom: Distribution (across time) of the time-dependent bias of estimates in E and I cell type. **(I)** Left: Distribution of time-averaged firing rates in E (top) and I neurons (bottom). Black traces are fits with log-normal distribution. Right: Distribution of coefficients of variation of interspike intervals for E and I neurons. **(J)** Distribution (across neurons) of time-averaged synaptic inputs to E (left) and I neurons (right). In E neurons, the distribution of inhibitory and of net synaptic inputs overlap. **(K)** Sum of synaptic inputs over time in a single E (top) and I neuron (bottom) in a simulation trial. **(L)** Distribution (across neurons) of Pearson’s correlation coefficients measuring the correlation of synaptic inputs in single E (red) and I (blue) neurons. For model parameters, see Table 1.

The network responds to M time-varying features of a sensory stimulus, $s_{k}\left(t\right)$ (e.g., for a visual stimulus, contrast, orientation, etc) received as inputs from an earlier sensory area (e.g., retina). We model features as independent Ornstein–Uhlenbeck (OU) processes (see Methods). The network’s objective is to compute a leaky integration of sensory features, a relevant computation of cortical sensory areas^46^. The target representations of the network, $x_{k}\left(t\right)$, are defined as

(1) $$\frac{dx_{k}(t)}{dt}=-\frac{1}{\tau }x_{k}(t)+s_{k}(t).$$

with τ a characteristic integration time-scale (Fig. 1A).

The network is composed of two neural populations of excitatory (E) and inhibitory (I) neurons, defined by their postsynaptic action which respects Dale’s law. For each population, $y\in \left\{E,I\right\}$, we define a population readout of each feature, ${\hat{x}}_{k}^{y}$, as a filtered weighted sum of spiking activity of neurons in the population,

(2) $$\frac{d{\hat{x}}_{k}^{y}(t)}{dt}=-\frac{1}{\tau }{\hat{x}}_{k}^{y}(t)+\sum_{i=1}^{N^{y}}w_{ki}^{y}f_{i}^{y}(t),$$

where $f_{i}^{y}\left(t\right)$ is the spike train of neuron i of type y and $w_{ki}^{y}$ are its decoding weights for features k=1,…M. As a result of the optimization, the decoding weights of the neurons are equivalent to neuron’s tuning parameters to the stimulus features (see Methods;^42^). We draw tuning parameters from a normal distribution with zero mean and SD $\sigma_{w}^{E}$ for E neurons and $\sigma_{w}^{I}$ for I neurons. We assume that every neuron encodes multiple (M>1) stimulus features^47^ and define the vector ${\text{w}}_{i}^{y}={\left[w_{1i}^{y},\ldots w_{Mi}^{y}\right]}^{⊤}$ as the tuning vector of neuron i (Fig. 1B).

Unlike previous approaches^28,48^, we hypothesize that E and I neurons have distinct normative objectives and define cell-type specific loss functions relative to the activity of the E and I neuron types. To implement at the same time, as requested by efficient coding, the constraints of faithful stimulus representation with limited computational resources^49^, we define the loss functions of E and I population as a weighted sum of a time-dependent encoding error and time-dependent metabolic cost:

(3) $$\begin{array}{cc} {\text{L}}^{y}(t)=\epsilon^{y}(t)+\beta \kappa^{y}(t), & y\in \{E,I\} \end{array}.$$

We refer to β, the parameter controlling the relative importance of the metabolic cost over the encoding error, as the metabolic constant of the network. We hypothesize that population readouts of E neurons, ${\hat{x}}_{k}^{E}\left(t\right)$, track the target representations, $x_{k}\left(t\right)$, and the population readouts of I neurons, ${\hat{x}}_{k}^{I}\left(t\right)$, track the population readouts of E neurons ${\hat{x}}_{k}^{E}\left(t\right)$, by minimizing the squared error between these quantities^37^ (see also^28,48^ for related approaches). Furthermore, we hypothesize the time-resolved metabolic cost to be proportional to the estimate of a momentary firing rate of the neural population. We thus define the variables of loss functions in Eq. 3 as

(4) $$\begin{array}{cc} \epsilon^{E}(t)=\sum_{k=1}^{M}{\left[x_{k}(t)-{\hat{x}}_{k}^{E}(t)\right]}^{2}, & \kappa^{E}(t)=\sum_{i=1}^{N^{E}}{\left[r_{i}^{E}(t)\right]}^{2},\\ \epsilon^{I}(t)=\sum_{k=1}^{M}{\left[{\hat{x}}_{k}^{E}(t)-{\hat{x}}_{k}^{I}(t)\right]}^{2}, & \kappa^{I}(t)=\sum_{i=1}^{N^{I}}{\left[r_{i}^{I}(t)\right]}^{2}, \end{array}$$

where $r_{i}^{y}y\in \left\{E,I\right\}$ is the low-pass filtered spike train of neuron i (single neuron readout) with time constant $\tau_{r}^{y}$. We then impose the following condition for spiking: a neuron shall emit a spike only if this decreases the loss function of its population in the immediate future. The condition for spiking also includes a noise term accounting for sources of stochasticity in spike generation^50^, including the effect of unspecific inputs from the rest of the brain.

By assuming that each neuron emits a spike at time t only if this decreases the loss function of its population (Eq. 3), we derived the dynamics and network structure of a spiking network that instantiates efficient coding (Fig. 1C, see Methods). The derived dynamics of the subthreshold membrane potential $V_{i}^{E}\left(t\right)$ and $V_{i}^{I}\left(t\right)$ obey the equations of the generalized leaky integrate and fire (gLIF) neuron

(5) $$\begin{array}{cc} \tau {\dot{V}}_{i}^{y}(t)=-\left(V_{i}^{y}(t)-V_{\text{rest}}^{y}\right)+R_{m}\left(I_{i}^{\text{syn},y}(t)-I_{i}^{\text{ad},y}(t)+I_{i}^{\text{ext},y}(t)\right), & y\in \{E,I\}, \end{array}$$

where $I_{i}^{\mathrm{syn},y}$, $I_{i}^{\mathrm{ad},y}$, and $I_{i}^{\mathrm{ext},y}$ are synaptic current, spike-triggered adaptation current and non-specific external current, respectively, $R_{m}$ is the membrane resistance and $V_{\mathrm{rest}}^{y}$ is the resting potential. This dynamics is complemented with a fire-and-reset rule: when the membrane potential reaches the firing threshold $\vartheta^{y}$, a spike is fired and $V_{i}^{y}\left(t\right)$ is set to the reset potential $V^{\mathrm{reset},y}$. The analytical solution in Eq. (5) holds for any number of neurons (with at least 1 neuron in each population) and predicts an optimal spike pattern to encode the presented external stimulus.

The synaptic currents in E neurons, $I_{i}^{\mathrm{syn},E}$, consist of feedforward currents, obtained as stimulus features $s_{k}\left(t\right)$ weighted by the tuning weights of the neuron, and of recurrent inhibitory currents. Synaptic currents in I neurons, $I_{i}^{\mathrm{syn},I}$, consist of recurrent excitatory and inhibitory currents (Fig. 1C).

The optimization of the loss function also yields structured recurrent connectivity (Fig. 1D). The synaptic strength between two neurons is proportional to their tuning similarity if the tuning similarity is positive; otherwise the synaptic weight is set to zero (Fig. 1E,F) to ensure that Dale’s law is respected. This also sets the overall connection probability to 0.5. (For a study of how efficient coding would be implemented if the above Dale’s law constraint was removed and each neuron is free to have either an inhibitory or excitatory effect depending on the postsynaptic target, see Supplementary Text 1). Neurons with opposite tuning have low connection probability, consistent with experimental results^51,52,53^ (Fig. 1D). Note that the structured recurrent connectivity leads to both E and I cells being stimulus-tuned, even though I cells do not receive feedforward inputs (Fig. 1C). The spike-triggered adaptation current of neuron i in population y, $I_{i}^{\mathrm{ad},y}$, has the dynamics of its low-pass filtered spike train $r_{i}^{y}\left(t\right)$. This current realizes spike-frequency adaptation or facilitation depending on the difference between the time constants of population and single neuron readout (see Results section “Weak spike-triggered adaptation optimizes network efficiency”). Finally, external currents have a constant mean, that depends on the parameter β, plus fluctuations that depend on the noise in the condition for spiking with intensity σ. Importantly, the relative weight of the metabolic cost over the encoding error controls the operating regime of the network biophysically plausibly, by modulating the mean of the external current. (Previous studies interpreted changes of the metabolic constant β as changes to the firing thresholds, which has less biophysical plausibility^36,33^) (see section “State-dependent coding and dynamics are controlled by the metabolic cost on spiking”).

To summarize, the analytical derivation of an optimally efficient network includes gLIF neurons^54,41,40,55,56^, a distributed code with mixed selectivity to the input stimuli, spike-triggered adaptation current, structured synaptic connectivity, and an operating regime controlled by the metabolic constant β.

The equations for the E-I network of gLIF neurons in Eq. (5) optimize the loss functions at any given time and for any set of parameters. In particular, the network equations have the same analytical form for any positive value of the metabolic constant β. To find a set of parameters that optimizes the overall performance, we defined a performance measure as the average over time and trials of the loss function. We then optimized the parameters by setting the metabolic constant β such that the encoding error weights 70 % and the metabolic error weights 30 % of the total performance, and by choosing all other parameters such as to minimize numerically our network performance measure (see Methods). The numerical optimization was performed by simulating a model of 400E and 100I units, a network size relevant for computations within one layer of a cortical microcolumn^57^. The set of model parameters that optimized network efficiency is detailed in Table 1. Unless otherwise stated, in all simulations we will use the optimal parameters of Table 1 and only vary those parameters detailed in the figure axes.

With optimally efficient parameters, population readouts closely tracked the target signals (Fig. 1G, M=3, $R^{2}=\left[0.95,0.97\right]$ for E and I neurons, respectively). When stimulated by our 3-dimensional time-varying feedforward input, the optimal E-I network provided a precise and unbiased estimator of the multi-dimensional and time-dependent target signal (Fig. 1H).

Next, we examined the emergent dynamical properties of an optimally efficient E-I network. The distribution of firing rates was well described by a log-normal distribution (Fig. 1I, left). Neurons fired irregularly, with mean coefficient of variation (CV) slightly smaller than 1 (Fig. 1I, right; $\mathrm{CV}=\left[0.97,0.95\right]$ for E and I neurons, respectively). We assessed E-I balance in single neurons through two complementary measures. First, we calculated the *average* (global) balance of E-I currents by taking the time-average of the net sum of currents^58^. Second, we evaluated the *instantaneous*^59^ (also termed detailed^45^) E-I balance using the Pearson correlation (ρ) of E and I currents received by a single neuron over time (see Methods).

We observed a strong average E-I balance (indicated by a net sum of synaptic inputs close to zero, with only a weak residual of inhibition in both E and I cells (Fig. 1J). Furthermore, we found a moderate instantaneous balance, stronger in I compared to E cell type (Fig. 1K-L, $\rho =\left[0.44,0.25\right]$, for I and E neurons, respectively). The presence of instantaneous balance between E and I synaptic currents within single neurons has been reported in cortical data^59,60^.

### Competition across neurons with similar stimulus tuning emerging in efficient spiking networks

We next explored coding properties emerging from recurrent synaptic interactions between E and I populations in the optimally efficient networks.

An approach that has recently provided empirical insight into local recurrent interactions between neurons is measuring the effective connectivity with cellular resolution, by photostimulating individual neurons and measuring the effect of such perturbation on other neurons in the network. Recent effective connectivity experiments photostimulated single E neurons in primary visual cortex and measured its effect on neighbouring neurons, finding that the photostimulation of an E neuron led to a decrease in firing rate of similarly tuned close-by neurons^61^. This effective lateral inhibition^26^ between E neurons with similar tuning to the stimulus implements competition between neurons for the representation of stimulus features (termed feature-specific competition^61^).

**Figure 2.**
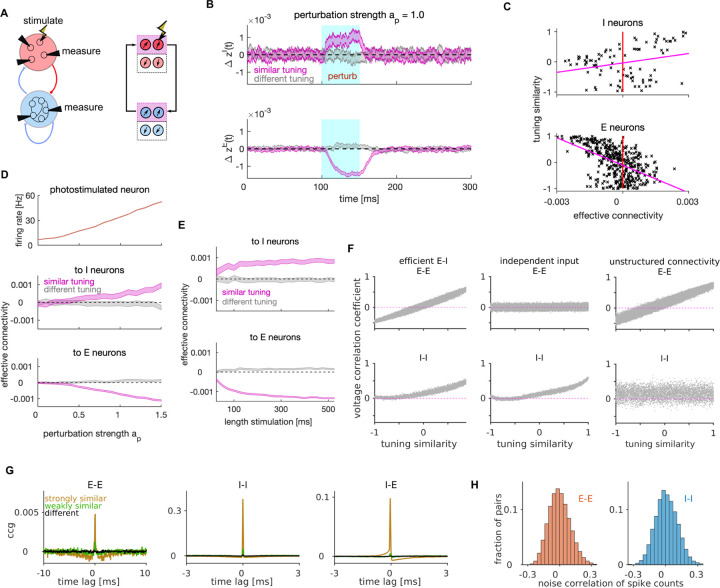
Mechanism of lateral excitation/inhibition in the efficient spiking network. **(A)** Left: Schematic of the E-I network and of the stimulation and measurement in a perturbation experiment. Right: Schematic of the propagation of the neural activity between E and I neurons with similar tuning. **(B)** Trial and neuron-averaged deviation of the firing rate from the baseline, for the population of I (top) and E (bottom) neurons with similar (magenta) and different tuning (gray) to the target neuron. The stimulation strength corresponded to an increase in the firing rate of the stimulated neuron by 28.0 Hz. **(C)** Scatter plot of the tuning similarity vs. effective connectivity to the target neuron. Red line marks zero effective connectivity and magenta line is the least-squares line. Stimulation strength was $a_{p}=1$. **(D)** Top: Firing rate of the photostimulated neuron as a function of the photostimulation strength. Middle: Effective connectivity with I neurons with similar and different tuning to the target neuron. Bottom: Effective connectivity with E neurons. **(E)** Effective connectivity with I (top) and E neurons (bottom) while varying the length of the stimulation window. The window for measuring the effective connectivity was always 50 ms longer than the stimulation window. **(F)** Correlation of membrane potentials vs. the tuning similarity in E (top) and I cell type (bottom), for the efficient E-I network (left), for the network where each E neuron receives independent instead of shared stimulus features (middle), and for the network with unstructured connectivity (right). In the model with unstructured connectivity, elements of each connectivity matrix were randomly shuffled. We quantified voltage correlation using the (zero-lag) Pearson’s correlation coefficient, denoted as $\rho \left(V_{i}^{y}\left(t\right),V_{j}^{y}\left(t\right)\right)$, for each pair of neurons. **(G)** Average cross-correlogram (CCG) of spike timing with strongly similar (orange), weakly similar (green) and different tuning (black). **(H)** Distribution of noise correlations across neuronal pairs. The correlation coefficient was measured in bins of 30 ms.

To assess how E-I interactions shape coding in efficient networks, we simulated photostimulation experiments in these networks. We performed such experiments in the absence of the feedforward input to insure all effects are only due to the recurrent processing and not to feedforward processing. We stimulated a randomly selected single “target” E neuron and measured the change in the instantaneous firing rate from the baseline firing rate, $\Delta z_{i}\left(t\right)$, in all the other I and E neurons (Fig. 2A, left). The photostimulation was modeled as an application of a constant depolarising current with a strength parameter, $a_{p}$, proportional to the distance between the resting potential and the firing threshold ($a_{p}=0$ means no stimulation, while $a_{p}=1$ indicates photostimulation at the firing threshold). We quantified the effect of the simulated photostimulation of a target E neuron on other E and I neurons, distinguishing neurons with either similar or different tuning with respect to the target neuron (Fig. 2A, right; Supplementary Fig. S2).

The photostimulation of the target E neuron increased the instantaneous firing rate of similarly-tuned I neurons and reduced that of other similarly-tuned E neurons (Fig. 2B, Supplementary Fig. S2). We quantified the effective connectivity as the difference between the time-averaged firing rate of the recorded cell in presence or absence of the photostimulation of the targeted cell, measured during perturbation and up to 50 ms after. We found positive effective connectivity on I and negative effective connectivity on E neurons with similar tuning to the stimulated neuron, with a positive correlation between tuning similarity and effective connectivity on I neurons and a negative correlation on E neurons (Fig. 2C). As we varied the strength of the photostimulation, the firing rate of the target neuron increased proportionally to the photostimulation strength, as did the effect of the perturbation on I and E neurons with similar tuning to the target neuron (Fig. 2D, Supplementary Fig. S2). As we varied the time window of photostimulation, we found that the effective connectivity converges within a time window of about 300 ms (Fig. 2E). We confirmed these effects of photostimulation in presence of a weak feedforward input (Supplementary Fig. S2), similar to the experiments of Ref^61^ in which photostimulation was applied during the presentation of visual stimuli with weak contrast.

In summary, lateral excitation of I neurons and lateral inhibition of E neurons with similar tuning is an emerging coding property of the efficient E-I network. Lateral excitation and inhibition leads to competition between neurons with similar tuning to stimulus features, comparable to that found in the visual cortex^61,62^. An intuitive summary of how this mechanism is implemented is that the E neuron that fires first activates I neurons with similar tuning. In turn, these I neurons inhibit all similarly tuned E neurons (Fig. 2A, right), preventing them to generate redundant spikes and encoding the sensory information that has already been encoded by the first spike. Suppression of redundant spiking allows efficient coding because it reduces the metabolic cost without compromising on encoded information^36^.

To explore further the consequences of E-I interactions for stimulus encoding, we next investigated the dynamics of lateral inhibition in a network driven by the feed-forward sensory input but without perturbing neurons. In this case, shared feedforward inputs $s_{k}\left(t\right)$ create a particular pattern of voltage correlations in E-E neuronal pairs, where voltage correlations linearly depend on the tuning similarity (Fig. 2F, left). The feedforward inputs are shared across neurons and weighted by the tuning parameters of E neurons. For this reason, they cause strong positive voltage correlations between E-E neuronal pairs with very similar tuning and strong negative correlations between pairs with very different (opposite) tuning (Fig. 2F, top-left). Voltage correlations between E-E pairs vanished regardless of tuning similarity when we made the inputs independent across neurons (Fig. 2F, top-middle), showing the relation between tuning similarity and voltage correlation occurs because of shared feedforward inputs. In contrast to E neurons, I neurons do not receive feedforward inputs and are driven only by similarly tuned E neurons (Fig. 2A, right). This causes positive voltage correlations in I-I neuronal pairs with similar tuning and vanishing correlations in neurons with different tuning (Fig. 2F, bottom-left). Such dependence of voltage correlations on tuning similarity disappears when removing the structure from the E-I synaptic connectivity (Fig. 2F, bottom-right).

Although membrane potentials could be strongly correlated or anti-correlated depending on tuning similarity (Fig. 2F, left), the coordination of spike timing of pairs of E neurons (measured with cross-correlograms or CCGs) was very weak (Fig. 2G-H). For I-I neuronal pairs, the peaks of CCGs were stronger than those observed in E-E pairs, but they were present only at very short lags (lags<1ms), and the same was true for E-I pairs. Additionally, noise correlations measured as Pearson correlation on spike counts in trials with the same stimulus ($r_{SC}$) had values distributed around zero (Fig. 2H). These findings lead to two conclusions. First, recurrent interactions of the efficient E-I network wipe away the effect of membrane potential correlations to produce largely uncorrelated spiking output, consistently with the efficient coding hypothesis of reducing redundancy in cases of low noise^3,6^. Second, such precise cancelling of correlations between voltages and the spiking output reflects the millisecond precision of information processing in efficient E-I networks.

### The effect of structured connectivity on coding efficiency and neural dynamics.

**Figure 3.**
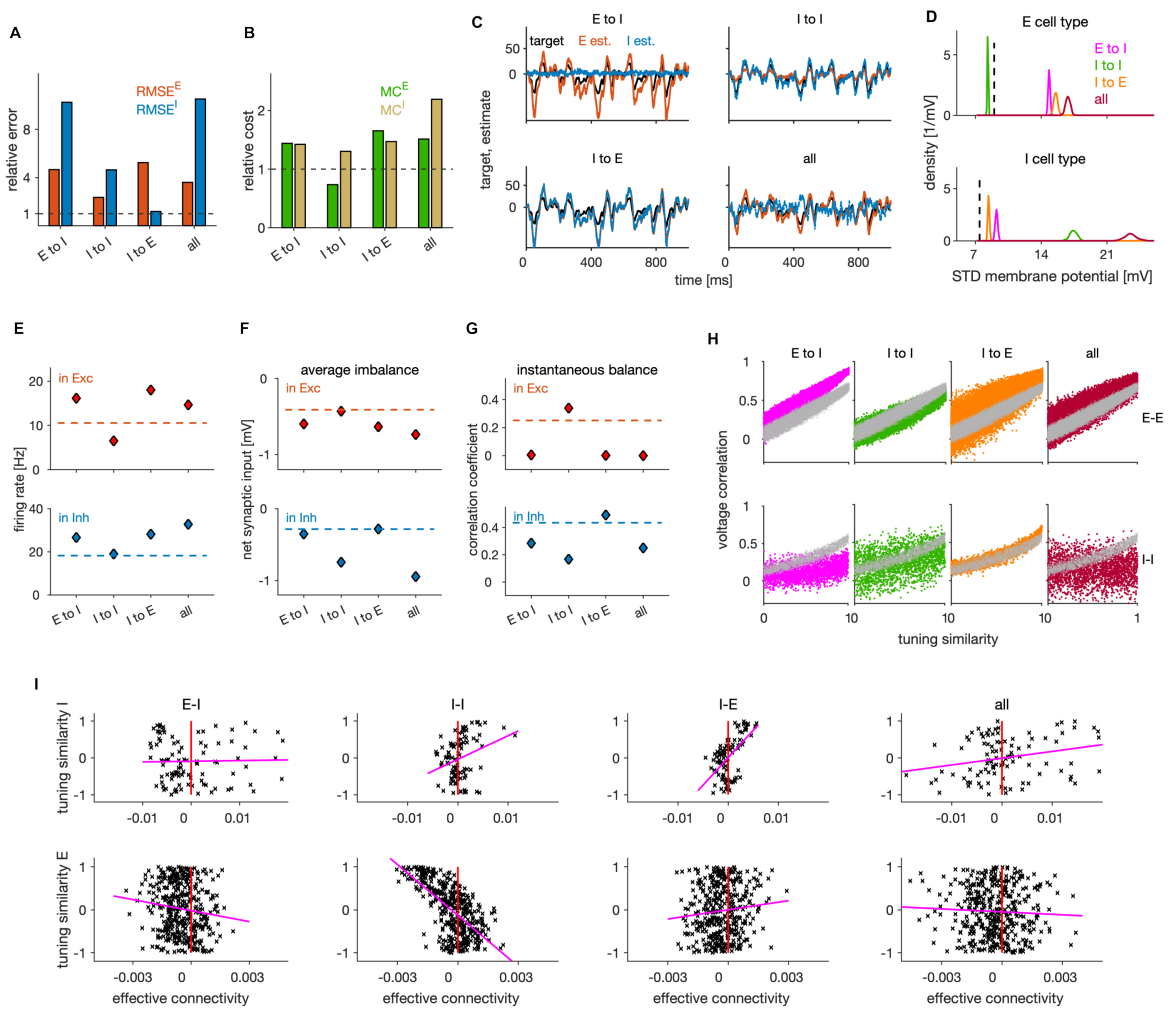
Effects of connectivity structure on coding efficiency, neural dynamics and lateral inhibition. **(A)** Relative error of networks with unstructured (shuffled) recurrent connectivity. The relative error is the RMSE of the unstructured network, relative to the RMSE of the structured network (dashed line). From left to right, we show the relative error for the unstructured E-I, I-I, I-E and all connectivities. **(B** Same as in **A**, showing the metabolic cost (MC) of unstructured networks relative to the metabolic cost of the structured network. **(C)** Target signal (black), E estimate (red) and I estimate (blue) in one particular input dimension, for networks with unstructured connectivity. **(D)** Standard deviation of the membrane potential (in mV) for networks with unstructured connectivity. Distributions are across neurons. The black vertical line marks the average SD of the structured network. **(E)** Average firing rate of E neurons (top) and I neurons (bottom), for different cases of unstructured networks. Dashed lines show the same measures for the structured case. **(F)** Same as in **E**, showing the average net synaptic input. **(G)** Same as in **E**, showing the time-dependent correlation of synaptic inputs. **(H)** Voltage correlation in E-E (top) and I-I neuronal pairs (bottom) for the four cases of unstructured connectivity (colored dots) and the equivalent result in the structured network (grey dots). We show the results for pairs with similar tuning. **(I)** Scatter plot of effective connectivity in I (top) and E neurons (bottom) versus tuning similarity to the stimulated (“target”) E neuron, for networks with unstructured connectivity. The magenta line is the least-squares regression line. The strength of the photostimulation is at threshold ($a_{p}=1.0$). Other parameters for all plots are in Table 1.

The analytical solution of the optimally efficient E-I network predicts that recurrent synaptic weights are proportional to the tuning similarity between neurons. We here investigated the role of such efficient connectivity structure by comparing the behavior of an efficiently structured network with a similar but randomly structured E-I network of the type studied in previous works^63,64,23^. We removed the connectivity structure by randomly permuting synaptic weights across neuronal pairs. We either randomized connections within a single connectivity type (E-I, I-I or I-E) or within all these three connectivity types at once (“all”). Such procedure destroys the relationship between tuning similarity and synaptic strength as in Fig. 1F while it preserves Dale’s law and the overall distribution of connectivity weights. We found that randomizing the connectivity structure significantly altered neural dynamics and coding (Fig. 3A-H). The structure in E-I and in I-E connectivity has a major effect on efficient coding. Randomizing E-I and I-E connectivity led to several-fold increases in the encoding error as well as to significant increases in the metabolic cost (Fig. 3A-B). In particular, with unstructured E-I connectivity the network failed completely to encode the target with I population (Fig. 3C).

Unstructured E-I and I-E connectivity also yielded an increase of the variance in the membrane potentials (Fig. 3D) and firing rate in E neurons (Fig. 3E), while pulling the average net synaptic inputs towards inhibition (Fig. 3F) and removing the instantaneous balance (Fig. 3G). Together, these findings suggest a shift from mean-driven to fluctuation-driven spiking activity as the connectivity structure is removed. The structure of E-I connectivity was also found to be crucial for the linear relation between voltage correlations and tuning similarity in pairs of I neurons (Fig. 3H, magenta). Interestingly, we found no effect of connectivity structure on the variability of spiking of single neurons, with both structured and unstructured networks showing strong variability (Supplementary Fig. S3), suggesting that the variability of spiking is independent of the connectivity structure.

Randomizing I-I connectivity was less detrimental to the coding efficiency as it led to a slightly higher encoding error, but to a lower metabolic cost, and still allowed for a relatively good tracking of target signals in both cell types (Fig. 3C, “permuted I to I”). Contrary to randomization of the E-I and I-E connectivity, shuffling I-I connectivity decreased the variance of the membrane potential, decreased the firing rate in E neurons and increased instantaneous balance in E neurons. Thus it had opposite effects compared to shuffling of E-I and I-E connectivity. To understand if there was a minimal connectivity structure necessary for efficient coding, we also removed the connectivity structure only partially, keeping like-to-like connectivity structure and removing all structure beyond like-to-like. This manipulation only had very modest effects on network’s coding and almost no effect on neural dynamics (Supplementary Fig. S3), thus showing that like-to-like structure of connectivity is largely sufficient to achieve efficient coding.

Finally, we analyzed how the structure in recurrent connectivity influences lateral inhibition that we observed in efficient (structured) networks (see Fig. 2A-E). We found that the dependence of lateral inhibition on tuning similarity vanish when the connectivity structure is fully removed (Fig. 3I, right), thus showing that connectivity structure is necessary for lateral inhibition. While networks with unstructured E-I and I-E connectivity still show inhibition in E neurons upon single neuron optostimulation (because of the net inhibitory effect of recurrent connectivity; Supplementary Fig. S4), this inhibition was largely unspecific to tuning similarity. Unstructured connectivity decreased the correlation between tuning similarity and effective connectivity from $r=\left[0.31,-0.54\right]$ in E and I neurons in a structured network to $r=\left[0.02,-0.13\right]$ and $r=\left[0.57,0.11\right]$ in networks with unstructured E-I and I-E connectivity, respectively (Fig. 3I, first and third from the left). Removing the structure in I-I connectivity, in contrast, increased the correlation between effective connectivity and tuning similarity in E neurons ($r=\left[0.30,-0.65\right]$, Fig. 3I, second from the left), showing that lateral inhibition takes place irrespective of the I-I connectivity structure. Furthermore, a partial removal of connectivity structure where we only removed the connectivity structure beyond like-to-like had smaller effects on lateral inhibition (Supplementary Fig. S4), thus confirming that like-to-like connectivity pattern is sufficient for lateral excitation/inhibition in I and E neurons.

While optimally structured connectivity predicted by efficient coding is biologically plausible, it may be difficult to realise it exactly on a synapse-by-synapse basis in biological networks. We verified the robustness of the model to small deviations from the optimal synaptic weights by adding a random jitter, proportional to the synaptic strength, to all synaptic connections (see Methods). The encoding performance and neural dynamics were barely affected by such perturbation, demonstrating that the network is robust against random perturbations of the optimal synaptic weights (Supplementary Fig. S3).

In summary, we found that some aspects of recurrent connectivity structure, such as the like-to-like organization of E-I and I-E connectivity, are crucial to achieve efficient coding. Instead, for other aspects there is considerable flexibility; the organization of I-I connectivity is less crucial, as is the connectivity structure beyond like-to-like, and adding small perturbations to optimal weights has only minor effects. Structured E-I and I-E, but not I-I connectivity, is necessary for a robust dependence of lateral inhibition on tuning similarity.

### Weak spike-triggered adaptation optimizes network efficiency

We next investigated the role of spike-triggered adaptation current, $I_{i}^{\mathrm{ad},y}$, that emerges from the optimally efficient solution (Eq. 5). This current provides a within-neuron feedback triggered by each spike, with time constant equal to that of the single neuron readout $\tau_{r}^{E}$ (E neurons) and $\tau_{r}^{I}$ (I neurons). The strength of the current is proportional to the difference in inverse time constants of single neuron and population readouts, $1/\tau -1/\tau_{r}^{y}$, and it is thus absent in previous studies assuming that these time constants are equal^29,28,33,31,44,42^.

**Figure 4.**
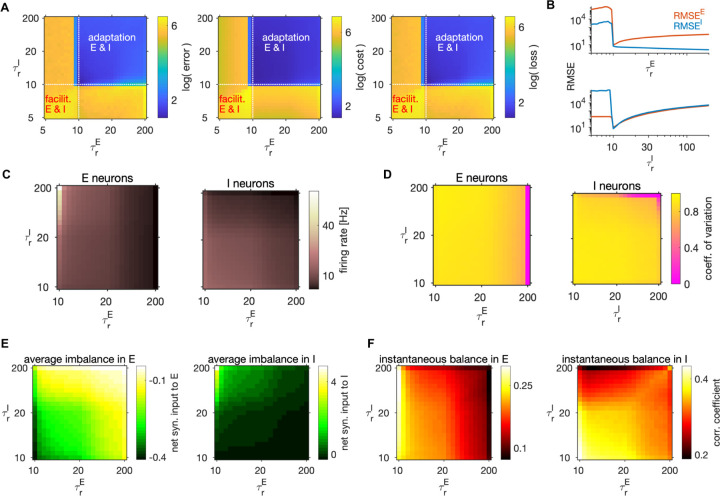
Adaptation, network coding efficiency and excitation-inhibition balance. **(A)** The encoding error (left), metabolic cost (middle) and average loss (right) as a function of single neuron time constants $\tau_{r}^{E}$ (E neurons) and $\tau_{r}^{I}$ (I neurons), in units of ms. These parameters set the sign, the strength, as well as the time constant of the feedback current in E and I neurons. Best performance is obtained in the top right quadrant, where the feedback current is spike-triggered adaptation in both E and I neurons. The performance measures are computed as a weighted sum of the respective measures across the E and I populations with equal weighting for E and I. All measures are plotted on the scale of the natural logarithm for better visibility. **(B)** Top: Log-log plot of the RMSE of the E (red) and the I (blue) estimates as a function of the time constant of the single neuron readout of E neurons, $\tau_{r}^{E}$. Feedback current in I neurons is set to 0. Bottom: Same as on the top, as a function of $\tau_{r}^{I}$ while the feedback current in E neurons is set to 0. **(C)** Firing rate in E (left) and I neurons (right), as a function of $\tau_{r}^{E}$ and $\tau_{r}^{I}$ in the regime with spike-triggered adaptation. **(D)** Same as in **(C)**, showing the coefficient of variation. **(E)** Average net synaptic input in E neurons (left) and in I neurons (right) as a function of $\tau_{r}^{E}$ and $\tau_{r}^{I}$. **(F)** Correlation coefficient of synaptic inputs to E (left) and I neurons (right) as a function of $\tau_{r}^{E}$ and $\tau_{r}^{I}$.

Depending on the sign of the difference of time constants, this spike-triggered current is negative, giving spike-triggered adaptation^39^, if the single-neuron readout has longer time constant than the population readout ($\tau_{r}^{y}>\tau$), or positive, giving spike-triggered facilitation, if the opposite is true ($\tau_{r}^{y}<\tau$) (Table 2). We expected that network efficiency would benefit from spike-triggered adaptation (in short, adaptation), because accurate encoding requires fast temporal dynamics of the population readouts, to capture fast fluctuations in the target signal, while we expect a slower dynamics in the readout of single neuron’s firing frequency, $r_{i}^{y}\left(t\right)$, a process that could be related to homeostatic regulation of single neuron’s firing rate^65,66^. Measuring performance of a simulated E-I network, we indeed found that optimal coding efficiency is achieved with weak adaptation in both cell types, and in particular in regimes where the adaptation is stronger in E compared to I neurons (Fig. 4A). We note that adaptation in E neurons promotes efficient coding because it enforces every spike to be error-correcting, while a spike-triggered facilitation in E neurons would lead to additional spikes that might be redundant and reduce network efficiency. Contrary to previously proposed model of adaptation in LIF neurons^38^, here strength and the time constant of adaptation are not independent, but they both depend on $\tau_{i}^{y}$, with larger $\tau_{i}^{y}$ yielding both longer and stronger adaptation.

To gain further insights on how adaptation influences network performance, we set the adaptation in one cell type to 0 and vary the strength of adaptation in the other cell type by varying the time constant of the single neuron readout. In the absence of adaptation in I neurons ($\tau_{r}^{I}=\tau$), adaptation in E neurons resulted in an increase of the encoding error in E neurons and a decrease in I neurons (Fig. 4B, top). Conversely, adaptation in I neurons (with no adaptation in E neurons) was harmful for the efficiency of the model, as it led to an increase in the encoding error in both cell types (Fig. 4B, bottom).

Firing rates and variability of spiking were sensitive to the strength of adaptation. As expected, adaptation in E neurons caused a decrease in the firing levels in both cell types (Fig. 4C). In contrast, adaptation in I neurons decreased the firing rate in I neurons, but increased the firing rate in E neurons, due to a decrease in the level of inhibition. Furthermore, adaptation decreased the variability of spiking, in particular in the cell type with strong adaptation (Fig. 4D), a well-known effect of spike-triggered adaptation in single neurons^67^.

### Instantaneous balance of synaptic currents predicts network efficiency better than the average E-I balance

Next, we tested the capability of instantaneous and average E-I balance to predict the efficiency of the network. Measuring average balance and instantaneous balance of synaptic inputs from electrophysiology recordings is possible^59,60,58^, while measuring efficiency from empirical data is challenging. The estimation of network efficiency requires the comparison between typically unknown network’s target representations and the population readouts. The estimation of the population readout, in turn, requires an estimation of decoding weights and the knowledge of spiking dynamics from a complete neural network.

We focused the analysis on regimes with adaptation, because these regimes gave better performance. In regimes with adaptation, time constants of single neuron readout influenced the average imbalance (Fig. 4E) as well as the instantaneous balance (Fig. 4F) in E and I cell type. The average balance was precise (with the net synaptic current close to 0) with strong adaptation in E neurons, and it got weaker when increasing the adaptation in I neurons (Fig. 4E). However, regimes with precise average balance in both cell types coincided with suboptimal efficiency (compare Fig. 4A, right and E).

To test how well the average imbalance and the instantaneous balance of synaptic inputs predict network efficiency, we concatenated the column-vectors of the measured average loss and of the average imbalance in each cell type and computed the Pearson correlation between these quantities. The correlation between the average imbalance and the average loss was weak in the E cell type (R=0.16) and close to zero in the I cell type (R=0.02), suggesting almost no relation between efficiency and average imbalance in the E cell type. In contrast, the average loss was negatively correlated with the instantaneous balance in both E (R=−0.35) and in I cell type (R=−0.45), showing that instantaneous balance of synaptic inputs is positively correlated with network efficiency.

When measured for varying levels of spike-triggered adaptation, unlike the average balance of synaptic inputs, the instantaneous balance is therefore a reliable predictor of network efficiency.

### State-dependent coding and dynamics are controlled by the metabolic cost on spiking

In our derivation of efficiency objectives, we obtained non-specific external current (in the following, non-specific current), described by the term $I_{i}^{\mathrm{ext},y}\left(t\right)$ and comprising mean and fluctuations (see Methods). Non-specific current captures the ensemble of all synaptic currents that are unrelated and un-specific with respect to the stimulus features. This non-specific term collates effects of synaptic currents from neurons untuned to the stimulus^68,69^, as well as synaptic currents from other brain areas. This term can also be conceptualized as the “background” synaptic activity that is thought to provide a large fraction of all synaptic inputs to both E and I neurons in cortical networks^70^, and which may modulate feedforward-driven responses by controlling how far is typically the membrane potential from the firing threshold^71^. Likewise, in our model, the external current does not directly convey information about the feedforward input features, but influences the operating regime of the network. The mean of the non-specific external currents is proportional to the metabolic constant β and its fluctuations reflect the noise that we assumed in the condition for spiking. Since β governs the trade-off between encoding error and metabolic cost (Eq. 3), higher values of β imply that more importance is assigned to the metabolic efficiency than to coding accuracy, yielding a reduction in firing rates. In the expression for the non-specific synaptic current, we found that the mean of the current is negatively proportional to the metabolic constant β (see Methods). The non-specific current is typically depolarizing, meaning that increasing β yields a weaker non-specific current and increases the distance between mean membrane potential and the firing threshold. Thus, an increase of the metabolic constant is expected to create a network state that is less responsive to the feedforward signal.

**Figure 5.**
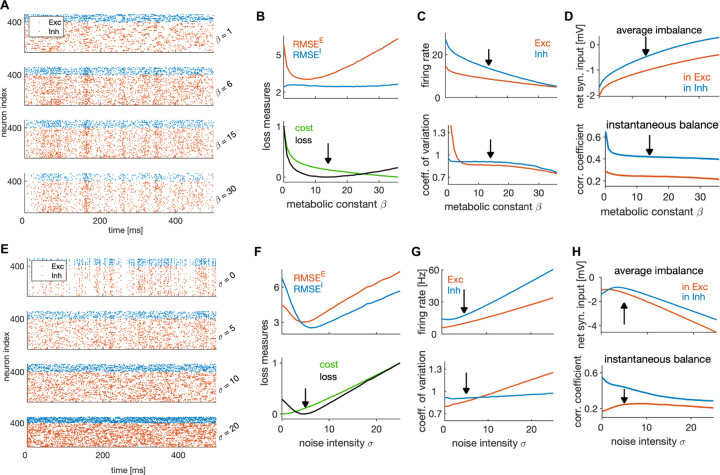
State-dependent coding and dynamics are controlled by non-specific currents. **(A)** Spike trains of the efficient E-I network in one simulation trial, with different values of the metabolic constant β. The network received identical stimulus across trials. **(B)** Top: RMSE of E (red) and I (blue) estimates as a function of the metabolic constant. Bottom: Normalized average metabolic cost and average loss as a function of the metabolic constant. Black arrow indicates the minimum loss and therefore the optimal metabolic constant. **(C)** Average firing rate (top) and the coefficient of variation of the spiking activity (bottom), as a function of the metabolic constant. Black arrow marks the metabolic constant leading to optimal network efficiency in **B**. **(D)** Average imbalance (top) and instantaneous balance (bottom) balance as a function of the metabolic constant. **(E)** Same as in **A**, but for different values of the noise intensity σ. **(F)** Same as in **B**, as a function of the noise intensity. The noise is a Gaussian random process, independent over time and across neurons. **(G)** Same as **C**, as a function of the noise intensity. **(H)** Top: Same as in **D**, as a function of the noise intensity. For plots in **B-D** and **F-H**, we computed and averaged results over 100 simulation trials with 1 second of simulation time. For other parameters, see Table 1.

We found the metabolic constant β to significantly influence the spiking dynamics (Fig. 5A). The optimal efficiency was achieved for non-zero levels of the metabolic constant (Fig. 5B). The metabolic constant modulated the firing rate as expected, with the firing rate decreasing with the increasing of the metabolic constant (Fig. 5C, top). It also modulated the variability of spiking, as increasing the metabolic constant decreased the variability of spiking in single neurons (Fig. 5C, bottom). Furthermore, it modulated the average imbalance and the instantaneous balance in opposite ways: larger values of β led to regimes that had stronger average balance, but weaker instantaneous balance (Fig. 5D). We note that, even with suboptimal values of the metabolic constant, the neural dynamics remained within biologically relevant ranges.

The fluctuation part of the non-specific current, modulated by the noise intensity σ, that we added in the definition of spiking rule for biological plausibility (see Methods), strongly affected the neural dynamics as well (Fig. 5E). The optimal performance was achieved with non-vanishing noise levels (Fig. 5F) and the beneficial effect of the noise in the non-specific current arose from its impact on the instantaneous E-I balance. While the average firing rate of both cell types, as well as the variability of spiking in E neurons, increased with noise variance (Fig. 5G), the average and instantaneous balance of synaptic currents exhibited a non-linear behavior as a function of noise variance (Fig. 5H). Due to decorrelation of membrane potentials by the noise, instantaneous balance decreased with increasing noise variance (Fig. 5H, bottom). Some level of noise in the non-specific inputs is therefore necessary to establish the optimal level of instantaneous E-I balance. Interestingly, single neurons manifest significant levels of spiking variability already in the absence of noise in the non-specific inputs (Fig. 5H, bottom), indicating that the recurrent network dynamics generates substantial variability even in absence of variability in the external current. Variability in absence of noise demonstrates the intrinsic chaotic behavior of the network^72^.

In summary, non-specific external currents derived in our optimal solution have a major effect on coding efficiency and on neural dynamics. The noise in the external current is particularly important to obtain optimal levels of the instantaneous E-I balance in I neurons.

### Optimal ratio of E-I neuron numbers and of the mean I-I to E-I synaptic efficacy coincide with biophysical measurements

Next, we investigated how coding efficiency and neural dynamics depend on the ratio of the number of E and I neurons ($N^{E}:N^{I}$ or E-I ratio) and on the relative synaptic strengths between E-I and I-I connections.

**Figure 6.**
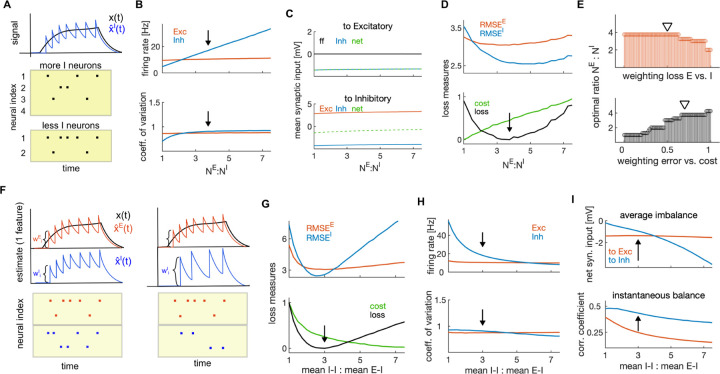
Optimal ratios of E-I neuron numbers and of mean I-I to E-I efficacy. **(A)** Schematic of the effect of changing the number of I neurons on firing rates of I neurons. As encoding of the stimulus is distributed among more I neurons, the number of spikes per I neuron decreases. **(B)** Average firing rate as a function of the ratio of the number of E to I neurons. Black arrow marks the optimal ratio. **(C)** Average net synaptic currents in E neurons (top) and in I neurons (bottom). **(D)** Top: Encoding error (RMSE) of the E (red) and I (blue) estimates, as a function of the ratio of E-I neuron numbers. Bottom: Same as on top, showing the cost and the average loss. Black arrow shows the minimum of the loss, indicating the optimal parameter. **(E)** Top: Optimal ratio of the number of E to I neurons as a function of the weighting of the average loss of E and I cell type (using the weighting of the error and cost of 0.7 and 0.3, respectively). Bottom: Same as on top, measured as a function of the weighting of the error and the cost when computing the loss. (The weighting of the losses of E and I neurons is 0.5.) Black triangles mark weightings that we typically used. **(F)** Schematic of the readout of the spiking activity of an E neuron (red) and an I neuron (blue) with equal amplitude of decoding weight (left) and with stronger decoding weight in the I neuron (right). Stronger decoding weight in the I neuron results in a stronger effect of spikes of the I neuron on the readout, leading to less spikes by the I neuron. **(G)** Same as in (D), as a function of the ratio of mean I-I to E-I efficacy. **(H)** Same as in **B**, as a function of the ratio of mean I-I to E-I efficacy. **(I)** Average imbalance (top) and instantaneous balance (bottom) balance, as a function of the ratio of mean I-I to E-I efficacy. For other parameters, see Table 1.

Efficiency objectives (Eq. 3) are based on population, rather than single-neuron activity. Our efficient E-I network thus realizes a computation of the target representation that is distributed across multiple neurons (Fig. 6A). We predict that, if number of neurons within the population decreases, neurons have to fire more spikes to achieve an optimal population readout because the task of tracking the target signal is distributed among fewer neurons. To test this prediction, we varied the number of I neurons while keeping the number of E neurons constant. As predicted, a decrease of the number of I neurons (and thus an increase in the ratio of the number of E to I neurons) caused a linear increase in the firing rate of I neurons, while the firing rate of E neurons stayed constant (Fig. 6B, top). However, the variability of spiking and the average synaptic inputs remained relatively constant in both cell types as we varied these ratios (Fig. 6B, bottom, C), indicating a compensation for the change in the ratio of E-I neuron numbers through adjustment in the firing rates. These results are consistent with the observation in neuronal cultures of a linear change in the rate of postsynaptic events but unchanged postsynaptic current in either E and I neurons for variations in the E-I neuron number ratio^73^.

The ratio of the number of E to I neurons had a significant influence on coding efficiency. We found a unique minimum of the encoding error of each cell type, while the metabolic cost increased linearly with the ratio of the number of E and I neurons (Fig. 6D). We found the optimal ratio of E to I neuron numbers to be in range observed experimentally in cortical circuits (Fig. 6D, bottom, black arrow, $N^{E}:N^{I}=3.75:1$;^74^). Due to the linear increase of the cost with the ratio of the number of E and I neurons (Fig. 6D, bottom, green), strong weighting of the error predicted higher ratios (Fig. 6E, bottom). Also the encoding error (RMSE) alone, without considering the metabolic cost, predicted optimal ratio of the number of E to I neurons within a plausible physiological range, $N^{E}:N^{I}=\left[3.75:1,5.25:1\right]$, with stronger weightings of the encoding error by I neurons predicting higher ratios (Fig. 6E, top).

Next, we investigated the impact of the strength of excitatory and inhibitory synaptic efficacy (EPSPs and IPSPs). In our model, the mean synaptic efficacy is fully determined by the distribution of tuning parameters (see Methods). As evident from the expression for the population readouts (Eq. 2), the amplitude of tuning parameters (which are also decoding weights) determines the amplitude of jumps of the population readout caused by spikes (Fig. 6F). The stronger the amplitude of these weights, the larger is the average impact of spikes on the population signals.

We parametrized the distribution of decoding weights as a normal distributions centered at zero, but allowed the standard deviation (SD) of distributions relative to E and I neurons ($\sigma_{w}^{E}$ and $\sigma_{w}^{I}$) to vary across E and I cell type. With such parametrization, we were able to analytically evaluate the mean E-I, I-I and I-E synaptic efficacy (see Methods). We found that in the optimally efficient network, the mean E-I and I-E synaptic efficacy is exactly balanced.

We next searched for the optimal ratio of the mean I-I to E-I efficacy as the parameter setting that maximizes network efficiency. Network efficiency was maximized when such ratio was about 3 to 1 (Fig. 6G). Our results predict the maximum E-I and I-E synaptic efficacy, averaged across neuronal pairs, of 0.75 mV, and the maximal I-I efficacy of 2.25 mV, values that are consistent with empirical measurements in the primary sensory cortex^75,52,53^.

Similarly to the ratio of E-I neuron numbers, a change in the ratio of mean E-I to I-E synaptic efficacy was compensated for by a change in firing rates, with stronger I-I synapses leading to a decrease in the firing rate of I neurons (Fig. 6H). Conversely, weakening the E-I and I-E synapses resulted in an increase in the firing rate in E neurons (Supplementary Fig. S5). This is easily understood by considering that weakening the E-I and I-E synapses activates less strongly the lateral inhibition in E neurons (Fig. 2) and thus leads to an increase in the firing rate of E neurons. We also found that single neuron variability remained almost unchanged when varying the ratio of mean I-I to E-I efficacy (Fig. 6H, bottom) and the optimal ratio corresponded with previously found optimal levels of average and instantaneous balance of synaptic inputs (Fig. 6I). The instantaneous E-I balance monotonically decreased with increasing ratio of I-I to E-I efficacy (Fig. 6I, bottom, Supplementary Fig. S5).

In summary, our analysis suggests that optimal coding efficiency is achieved with four times more E neurons than I neurons and with mean I-I synaptic efficacy about 3 times stronger than the E-I and I-E efficacy. The optimal network has less I than E neurons, but the impact of spikes of I neurons on the population readout is stronger, also suggesting that spikes of I neurons convey more information.

### Dependence of efficient coding and neural dynamics on the timescales and dimensionality of the stimulus

**Figure 7.**
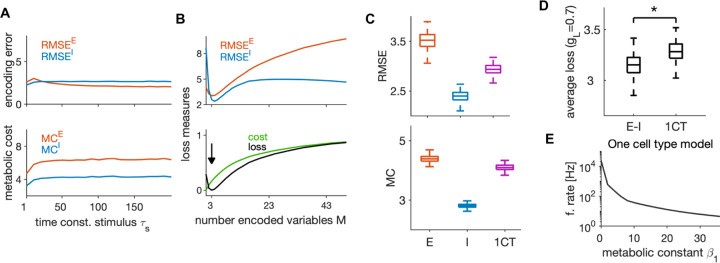
Dependence of efficient coding and neural dynamics on stimulus parameters and advantages of E-I versus one cell type model architecture. **(A)** Top: Root mean squared error (RMSE) of E estimates (red) and I estimates (blue), as a function of the time constant of stimulus features. Bottom: Same as on top, showing the metabolic cost (MC) of E and I cell type. The time constant $\tau_{s}$ is the same for all stimulus features. **(B)** Top: Same as in **A** top, measured as a function of the number of stimulus features M. Bottom: Normalized cost and the average loss as a function of the number of input features. Black arrow marks the minimum loss and the optimal parameter M. **(C)** Root mean squared error (top) and metabolic cost (bottom) in E and I populations in the E-I model and in the 1CT model. The distribution is across simulation trials. **(D)** Average loss in the E-I and 1CT models with weighting $g_{L}=0.7$ for the error (and 0.3 for the cost). **(E)** Firing rate in the 1CT model as a function of the metabolic constant. For other parameters of the E-I model see Table 1, and for the 1CT model see Supplementary Table S1.

We finally investigated how the network’s behavior depends on the timescales and dimensionality of the input stimulus features. We manipulated the stimulus timescales by changing the time constant of the Ornstein-Uhlenbeck (O-U) process. The network efficiently encoded stimulus features when their time constants varied between 1 and 200 ms, with stable encoding error, metabolic cost (Fig. 7A) and neural dynamics (Supplementary Fig. S6).

Finally, we tested how the network’s behavior changed when we varied the number of stimulus features M processed by the network. The encoding error of E (${\mathrm{RMSE}}^{E}$) and I neurons (${\mathrm{RMSE}}^{I}$) had a minimum at 3 and 4 stimulus features, respectively (Fig.7B, top), while the metabolic cost increased monotonically with the number of features (Fig.7B, bottom). The number of features that optimized network efficiency (the average loss) ranged between $M=\left[1,4\right]$. With strong weighting of the error ($g_{L}\geq 0.89$), the optimal number of features was M=4, and with strong weighting of the cost, ($g_{L}<0.27$), the optimal number of features was M=1. It is intriguing that the optimal encoding performance, when assuming the weighting for the error is stronger than for the cost, is achieved not for a single stimulus feature, but for 3 or 4 independent features. Increasing the number of features beyond the optimal number resulted in a monotonic increase in firing rates for both cell types and in a contrasting effect on average and instantaneous balance, as it increased the average E-I balance and weakened the instantaneous balance (Supplementary Fig. S6).

In sum, we found the optimal network efficiency in presence of several (3 or 4) stimulus features, and a surprising ability of the network to accurately encode stimuli on a wide range of timescales.

### Advantages of E-I versus one cell type model architecture for coding efficiency and robustness to parameter variations

Neurons in the brain are either excitatory or inhibitory. To understand how differentiating E and I neurons benefits efficient coding, we compared the properties of our efficient E-I network with an efficient network with a single cell type (1CT). The 1CT model is a simplification of the E-I model (see Supplementary Text 1) and has been derived and analyzed in previous studies^29,28,36,33,44,42^. We compared the average encoding error (RMSE), the average metabolic cost (MC), and the average loss (see Supplementary Text 2) of the E-I model against the one cell type (1CT) model. Compared to the 1CT model, the E-I model exhibited a higher encoding error and metabolic cost in the E population, but a lower encoding error and metabolic cost in the I population (Fig. 7C). The average loss of the E-I model was significantly smaller than that of the 1CT model when using the typical weighting of the error and the cost of $g_{L}=0.7$ (Fig. 7D), as well as for the vast majority of other weightings ($g_{L}\leq 0.95$; Supplementary Fig. S1).

We further compared the 1CT and E-I models in terms of the robustness of firing rates to changes in the metabolic constant. Consistently with previous studies^36,35^, firing rates in the 1CT model were highly sensitive to variations in the metabolic constant (Fig. 7E, note the logarithmic scale on the y-axis), with a superexponential growth of the firing rate with the inverse of the metabolic constant in regimes with metabolic cost lower than optimal. This is in contrast to the E-I model, whose firing rates exhibited lower sensitivity to the metabolic constant, and never exceeded physiological limits (Fig. 5C). Because our E-I model does not incorporate a saturating input-output function as in^34^ that would constrain the range of firing rates, the ability of the E-I model to maintain firing rates within biologically plausible limits emerges as a highly desirable dynamic property.

In summary, we found that the optimal E-I model is more efficient than the 1CT model. Beyond the performance of optimal models, the E-I model is advantageous with respect to the 1CT model also because it does not enter into states of physiologically unrealistic firing rates.

## Discussion

We analyzed comprehensively the structural, dynamical and coding properties that emerge in networks of spiking neurons that implement optimally the principle of efficient coding. We demonstrated that efficient recurrent E-I networks form highly accurate and unbiased representations of stimulus features with biologically plausible parameters, biologically plausible neural dynamics, instantaneous E-I balance and like-to-like lateral inhibition. The network can implement efficient coding with stimulus features varying over a wide range of timescales and when encoding even multiple such features. Here we discussed the implications of these findings.

By a systematic study of the model, we determined the model parameters that optimize network efficiency. Strikingly, the optimal parameters (including the ratio between the number of E and I neurons, the ratio of I-I to E-I synaptic efficacy and parameters of non-specific currents) were consistent with parameters measured empirically in cortical circuits, and generated plausible spiking dynamics. This result lends credibility to the hypothesis that cortical networks might be designed for efficient coding and may operate close to optimal efficiency, as well as provides a solid intuition about what specific parameter ranges (e.g. higher numbers of E and than I neurons) may be good for. Efficient networks still exhibited realistic dynamics and reasonably efficient coding in the presence of moderate deviations from the optimal parameters, suggesting that the optimal operational point of such networks is relatively robust. We also found that optimally efficient analytical solution derives generalized LIF (gLIF) equations for neuron models^37^. While gLIF^67,40^ and LIF^63,64^ models are reasonably biologically plausible and are widely used to model and study spiking neural network dynamics, it was unclear how their parameters affect network-level information coding. Our study provides a principled way to determine uniquely the parameter values of gLIF networks that are optimal for efficient information encoding. Studying the dynamics of gLIF networks with such optimal parameters thus provides a direct link between optimal coding and neural dynamics. Moreover, our formalism provides a framework for the optimization of neural parameters that can in principles be used not only for neural network models that study brain function but also for the design of artificial neuromorphic circuits that perform information coding computations^76,77^.

Unlike in previous randomly-connected recurrent networks of LIF and gLIF spiking neurons,^63,64^ in our efficient-coding solution, a highly structured E-I, I-I and I-E synaptic connectivity emerges as an optimal structural solution to support efficient coding. Our model generates a number of insights about the role of structured connectivity in efficient information processing. A first insight is that I neurons develop stimulus feature selectivity because of the structured recurrent connectivity. This is in line with recent reports of stimulus feature selectivity of inhibitory neurons, including in primary visual cortex^78,79,80^. A second insight is that a network with structured connectivity shows stronger average and instantaneous E-I balance, as well as significantly lower variance in membrane potentials compared to an equivalent network with the same connections organized randomly. This implies that the connectivity structure determines the operating regime of the network. In particular, a network structured as in our efficient coding solution operates in a dynamical regime that is more stimulus-driven, compared to an unstructured network that is more fluctuation driven. A third insight is that the structured network exhibits a several-fold lower encoding error compared to unstructured networks and achieves this precision with lower firing rates. Network with structured recurrent connectivity creates more precise representations with less spikes and is therefore significantly more efficient compared to unstructured networks. Our analysis of the effective connectivity created by the efficient connectivity structure shows that this structure sharpens stimulus representations, reduces redundancy and increases metabolic efficiency by implementing feature-specific competition, that is a negative effective connectivity between E neurons with similar stimulus tuning, as proposed by recent theories^30^ and experiments^61,62^ of computations in visual cortex.

Our perturbation experiments on single E neurons predict a negative like-to-like effective connectivity between E neurons with similar tuning, as found experimentally in the mouse primary visual cortex with 2-photon optogenetic perturbations of E neurons^61,62^. This suggests that the effective connectivity found in mouse visual cortex could reflect efficient coding in visual cortex. Comparing effective connectivity in models and experiments is also useful for ruling in and out different theories of how efficient coding may be implemented in primary visual cortex. Earlier theories^4,11^ found evidence for efficient coding in visual cortex and proposed that such efficient computations relied only on feedforward connectivity; thus they predicted null effective connectivity between visual neurons and were ruled out by the empirical effective connectivity measures^61,62^. Our model, instead, implements efficient coding with recurrent interactions, suggesting a mechanism that is compatible with these empirical measures. Importantly, we made predictions for further optogenetics experiments that could better constraints models of visual cortical efficient coding. Previous studies^61^ optogenetically stimulated E neurons but did not determine whether the recorded neurons where excitatory or inhibitory. Our model predicts that stimulation of E neurons would increase firing in similarly tuned I neurons and decrease firing in similarly tuned E neurons. Our analysis confirms earlier model predictions^81^ that like-to-like connectivity between E and I neurons is necessary for lateral inhibition and competition between E neurons. Beyond like-to-like connectivity, our model predicts an optimally efficient connectivity where synaptic strength positively correlates with pair-wise tuning similarity, a connectivity pattern that was recently observed experimentally^82^.

Our study determines how structured E-I connectivity affects the dynamics of E-I balancing and how this relates to information coding. Previous work^32^ proposed that the E-I balance in efficient spiking networks operates on a finer time scale than in classical balanced E-I networks with random connectivity^64^. However, a theory to determine the exact levels of instantaneous E-I balance that is optimal for coding was lacking. Consistent with the general idea put forth in^32,31,48^, we here showed that moderate levels of E-I balance are optimal for coding, and that too strong levels of instantaneous E-I balance are detrimental to coding efficiency. Our results predict that like-to-like structured E-I-E connectivity is necessary for optimal levels of temporal E-I balance. Finally, the E-I-E structured connectivity that we derived supports optimal levels of instantaneous E-I balance and causes desynchronization of the spiking output. Such intrinsically generated desynchronization is a desirable network property that in previously proposed models could only be achieved by the less plausible addition of strong noise to each neuron^31,35^.

We found that our efficient network, optimizing the representation of a leaky integration of stimulus features, does not require recurrent E-E connections. Supporting this prediction, recurrent E-E connections were reported to be sparse in primary visual cortex^83^), and the majority of E-E synapses in the visual cortex were suggested to be long-range^84^. However, future studies could address the role of recurrent excitatory synapses, that were shown to emerge in efficient coding networks implementing computations beyond leaky integration such as linear mixing of features^37^. Efficient networks with E-E connectivity show neural dynamics that goes well beyond the canonical case analyzed here and can potentially describe persistent network dynamics^44^. Such networks would also allow to address whether biologically plausible efficient networks exhibit criticality, as suggested by^85^. Finally, we note that efficient encoding might be the primary normative objective in sensory areas, while areas supporting high-level cognitive tasks such as decision-making might include other computational objectives such as efficient transmission of information downstream to generate reliable behavioral outputs^86,87,88,25^.

## Methods

### Overview of the current approach and of differences with previous approaches

In the following, we present a detailed derivation of the E-I spiking network implementing the efficient coding principle. The analytical derivation is based on previous works on efficient coding with spikes^28,36^, and in particular on our recent work^37^. While these previous works analytically derived feedforward and recurrent transmembrane currents in leaky integrate-and fire neuron models, these models did not contain any synaptic current unrelated to feedforward and recurrent processing. Non-specific synaptic current was suggested to be important for an accurate description of coding and dynamics in cortical networks^71^. In the model derivation that follows, we also derived non-specific external current from efficiency objectives.

As we mapped the efficient coding objective on biologically plausible neural implementations, we found that such implementations (with plausible biophysical parameters) requires a transmembrane current that is independent of feedforward and recurrent processing. We interpreted this current as non-specific external current (shortly, non-specific current), collating the ensemble of synaptic projections from other brain areas that are not directly involved in processing of feedforward stimulus features^70^, as well as synaptic inputs from the local network from neurons that are not tuned to feedforward stimulus features^69^. The mechanistic effect of the non-specific current is to regulate the distance to firing threshold, a role that is close to the notion of “background” synaptic activity in cortical neurons^71^.

Moreover, previous models on efficient coding did not thoroughly consider physical units of variables that were interpreted as biophysical quantities (such as membrane potentials, firing thresholds, etc.). As these biophysical variables were derived from computational variables (such as target signals and population readouts), it remained unclear how biophysical variables might acquire their physical units. Here, we assigned physical units to the computational variables and thus naturally endowed the model with physical units. The network developed here allows for a better compatibility of efficient spiking models with neurobiology compared to previous works on efficient coding with spikes. With this model, we aim to describe neural dynamics and computation in early sensory cortices such as the primary visual cortex in rodents, even though many principles of the model developed here could be relevant throughout the brain.

### Introducing variables of the model

We consider two types of neurons, excitatory neurons E and inhibitory neurons I. We denote as $N^{E}$ and $N^{I}$ the number of E-cells and I-cells, respectively. The spike train of neuron i of type $y\in \left\{E,I\right\}$, $i=1,2,\ldots ,N^{y}$, is defined as a sum of Dirac delta functions,

(6) $$f_{i}^{y}(t)=\sum_{\alpha }\delta \left(t-t_{i}^{y,\alpha }\right),$$

where $t_{i}^{y,\alpha }$ is the time of the a-th spike of that neuron, defined as a time point at which the membrane potential of neuron i crosses the firing threshold.

We define the readout of the spiking activity of neuron i of type y (in the following, “single neuron readout”) as a leaky integration of its spike train,

(7) $$\begin{array}{cc} \frac{dr_{i}^{y}(t)}{dt}=-\lambda_{r}^{y}r_{i}^{y}(t)+f_{i}^{y}(t), & y\in \{E,I\}, \end{array}$$

with $\lambda_{r}$ denoting the inverse time constant. This way, the quantity ${\tilde{r}}_{i}^{y}\left(t\right)=\lambda_{r}^{y}r_{i}^{y}\left(t\right)$ represents an estimate of the instantaneous firing rate of neuron i.

We denote as $s_{k}\left(t\right)$, k=1,2,…,M the set of M dynamical features of the external stimulus (in the following, stimulus features) which are transmitted to the network through a feedforward sensory pathway. The stimulus features have the unit of the square root of millivolt, ${\left(mV\right)}^{\frac{1}{2}}$. The k-th dimension of the target signal is then obtained through a leaky integration of the feedforward variable, $s_{k}\left(t\right)$^29^, with inverse time constant λ, as

(8) $$\frac{dx_{k}(t)}{dt}=-\lambda x_{k}(t)+s_{k}(t).$$

Furthermore, we define a linear population readout of the spiking activity of E and I neurons

(9) $$\begin{array}{cc} \frac{d{\hat{x}}_{k}^{y}(t)}{dt}=-\lambda {\hat{x}}_{k}^{y}(t)+\sum_{i=1}^{N^{y}}w_{ki}^{y}f_{i}^{y}(t), & y\in \{E,I\}, \end{array}$$

with $w_{ki}^{y}$ in units of ${\left(mV\right)}^{\frac{1}{2}}$. Here, each neuron i of type y is associated with a vector ${\text{w}}_{i}^{y}:={\left[w_{1i}^{y},\ldots ,w_{Mi}^{y}\right]}^{⊤}$ of M tuning parameters representing the readout weight of neuron i with respect to the M population readouts in Eq. 9. These readout weights can be combined in the $M\times N^{y}$ matrix $W^{y}=\left[w_{ki}^{y}\right]$. The rows of this matrix define the patterns of readout weights ${\tilde{\text{w}}}_{k}^{y}:={\left[w_{k1}^{y},\ldots ,w_{kN^{y}}^{y}\right]}^{⊤}$ for each signal dimension k=1,2,…,M.

### Loss functions

We assume that the activity of a population y∈{E,I} is set so as to minimize a time-dependent encoding error and a time-dependent metabolic cost:

(10) $${\text{L}}^{y}(t)=\epsilon^{y}(t)+\beta^{y}\kappa^{y}(t),$$

with $\beta^{y}>0$ in units of mV the Lagrange multiplier which controls the weight of the metabolic cost relative to the encoding error. The time-dependent encoding error is defined as the squared distance between the targets and their estimates, and the role of estimates is assigned to the population readouts ${\hat{x}}_{k}^{y}\left(t\right)$. In E neurons, the targets are defined as the target signals $x_{k}\left(t\right)$, and their estimators are the population readouts of the spiking activity of E neurons, ${\hat{x}}_{k}^{E}\left(t\right)$. In I neurons, the targets are defined as the population readouts of E neurons ${\hat{x}}_{k}^{E}\left(t\right)$ and their estimators are the population readouts of I neurons ${\hat{x}}_{k}^{I}\left(t\right)$. Furthermore, the time-dependent metabolic cost is proportional to the squared estimate of the instantaneous firing rate, summed across neurons from the same population. Following these assumptions, we define the variables of loss functions in Eq. 10 as

(11) $$\begin{array}{l} \begin{array}{cc} \epsilon^{E}(t)=\sum_{k=1}^{M}{\left[x_{k}(t)-{\hat{x}}_{k}^{E}(t)\right]}^{2}, & \kappa^{E}(t)=\sum_{i=1}^{N^{E}}{\left[r_{i}^{E}(t)\right]}^{2}, \end{array}\\ \begin{array}{cc} \epsilon^{I}(t)=\sum_{k=1}^{M}{\left[{\hat{x}}_{k}^{E}(t)-{\hat{x}}_{k}^{I}(t)\right]}^{2}, & \kappa^{I}(t)=\sum_{i=1}^{N^{I}}{\left[r_{i}^{I}(t)\right]}^{2}. \end{array} \end{array}$$

We use a quadratic metabolic cost because it promotes the distribution of spiking across neurons^28^. In particular, the loss function of I neurons, $L^{I}\left(t\right)$ implies the relevance of the approximation: ${\hat{x}}_{k}^{E}\left(t\right)\approx {\hat{x}}_{k}^{I}\left(t\right)$ (see $\epsilon^{I}$ in the Eq. 11), which will be used in what follows.

### When shall a neuron spike?

We minimize the loss function by positing that neuron i of type y∈{E,I} emits a spike as soon as its spike decreases the loss function of its population y in the immediate future^37^. We also define $t^{-}$ and $t^{+}$ as the left- and right-sided limits of a spike time $t=t_{i}^{y,\alpha }$, respectively. Thus, at the spike time, the following jump condition must hold:

(12) $$\begin{array}{cc} L^{y}\left(t^{+}\right)\leq L^{y}\left(t^{-}\right)+\xi_{i}^{y}\left(t^{-}\right), & y\in \{E,I\}, \end{array}$$

with $\xi_{i}^{y}$ in units of mV. Here, the arguments $t^{-}$ and $t^{+}$ denote the left- and right-sided limits of the respected functions at time t. Furthermore, we added a noise term on the right-hand side of the Eq. (12) in order to consider the stochastic nature of spike generation in biological networks^50^. A convenient choice for the noise $\xi_{i}^{y}\left(t\right)$ is the Ornstein-Uhlenbeck process obeying

(13) $${\dot{\xi }}_{i}^{y}(t)=-\lambda \xi_{i}^{y}(t)+\sqrt{2\lambda }\sigma_{\xi }^{y}\eta_{i}^{y}(t),$$

where $\eta_{i}^{y}$ is a Gaussian white noise with auto-covariance function $\langle \eta_{i}(t)\eta_{j}\left(t^{\prime }\right)\rangle =\delta_{ij}\delta \left(t-t^{\prime }\right)$. The process $\xi_{i}^{y}\left(t\right)$ has zero mean and auto-covariance function $\langle \xi_{i}(t)\xi_{j}\left(t^{\prime }\right)\rangle ={\left(\sigma_{\xi }^{y}\right)}^{2}\delta_{ij}e^{-\lambda \left|t-t^{\prime }\right|}$, with ${\left(\sigma_{\xi }^{y}\right)}^{2}$ the variance of the noise.

By applying the condition for spiking in Eq. (12) using y=E and y=I, respectively, we get

(14) $$\begin{array}{l} \left[\sum_{k=1}^{M}{\left(x_{k}\left(t^{+}\right)-{\hat{x}}_{k}^{E}\left(t^{+}\right)\right)}^{2}+\beta^{E}\sum_{j=1}^{N^{E}}{\left(r_{j}^{E}\left(t^{+}\right)\right)}^{2}\right]-\left[\sum_{k=1}^{M}{\left(x_{k}\left(t^{-}\right)-{\hat{x}}_{k}^{E}\left(t^{-}\right)\right)}^{2}+\beta^{E}\sum_{j=1}^{N^{E}}{\left(r_{j}^{E}\left(t^{-}\right)\right)}^{2}\right]\leq \xi_{i}^{E}\left(t^{-}\right),\\ \left[\sum_{k=1}^{M}{\left({\hat{x}}_{k}^{E}\left(t^{+}\right)-{\hat{x}}_{k}^{I}\left(t^{+}\right)\right)}^{2}+\beta^{I}\sum_{j=1}^{N^{I}}{\left(r_{j}^{I}\left(t^{+}\right)\right)}^{2}\right]-\left[\sum_{k=1}^{M}{\left({\hat{x}}_{k}^{E}\left(t^{-}\right)-{\hat{x}}_{k}^{I}\left(t^{-}\right)\right)}^{2}+\beta^{I}\sum_{j=1}^{N^{I}}{\left(r_{j}^{I}\left(t^{-}\right)\right)}^{2}\right]\leq \xi_{i}^{I}\left(t^{-}\right). \end{array}$$

According to the definitions in Eqs. (7) and (9), if neuron i fires a spike at time $t=t_{i}^{y,\alpha }$, it causes a jump of its own filtered spike train (but not of other neurons j≠i), as well as of the population readout of the population it belongs to. Therefore, when neuron i fires a spike, we have for a given neuron j and a given population readout k:

(15a) $$r_{j}^{y}\left(t^{+}\right)=r_{j}^{y}\left(t^{-}\right)+\delta_{ij},$$

(15b) $${\hat{x}}_{k}^{y}\left(t^{+}\right)={\hat{x}}_{k}^{y}\left(t^{-}\right)+w_{ki}^{y}.$$

By inserting Eq. (15a)-(15b) in Eq. (12), we find that neuron i of type y should fire a spike if the following condition holds:

(16a) $$\begin{array}{l} \sum_{k=1}^{M}\left\{w_{ki}^{E}\left(x_{k}(t)-{\hat{x}}_{k}^{E}(t)\right)\right\}-\beta^{E}r_{i}^{E}(t)\geq \frac{1}{2}\left(\sum_{k=1}^{M}{\left(w_{ki}^{E}\right)}^{2}+\beta^{E}-\xi_{i}^{E}(t)\right),\\ \sum_{k=1}^{M}\left\{w_{ki}^{I}\left({\hat{x}}_{k}^{E}(t)-{\hat{x}}_{k}^{I}(t)\right)\right\}-\beta^{I}r_{i}^{I}(t)\geq \frac{1}{2}\left(\sum_{k=1}^{M}{\left(w_{ki}^{I}\right)}^{2}+\beta^{I}-\xi_{i}^{I}(t)\right). \end{array}$$

These equations tell us when the neuron i of type E and I, respectively, emits a spike, and are similar to the ones derived in previous works^37,28^. In addition to what has been found in these previous works, we here also find that each term on the left- and right-hand side in the Eq 16a has the physical units of millivolts.

We note that the expression derived from the minimization of the loss function of E neurons in the top row of Eq. (16a) is independent of the activity of I neurons, and would thus lead to the E population being unconnected with the I population. In order to derive a recurrently connected E-I network, the activity of E neurons must depend on the activity of I neurons. We impose this property by using the approximation of estimates that holds under the assumption of efficient coding in I neurons (see $\epsilon^{I}$ in the Eq. 11), ${\hat{x}}_{k}^{I}\left(t\right)\approx {\hat{x}}_{k}^{E}\left(t\right)\forall k=1\ldots ,M$. This yields the following conditions:

(16b) $$\begin{array}{l} \sum_{k=1}^{M}\left\{w_{ki}^{E}\left(x_{k}(t)-{\hat{x}}_{k}^{I}(t)\right)\right\}-\beta^{E}r_{i}^{E}(t)\geq \frac{1}{2}\left(\sum_{k=1}^{M}{\left(w_{ki}^{E}\right)}^{2}+\beta^{E}-\xi_{i}^{E}(t)\right),\\ \sum_{k=1}^{M}\left\{w_{ki}^{I}\left({\hat{x}}_{k}^{E}(t)-{\hat{x}}_{k}^{I}(t)\right)\right\}-\beta^{I}r_{i}^{I}(t)\geq \frac{1}{2}\left(\sum_{k=1}^{M}{\left(w_{ki}^{I}\right)}^{2}+\beta^{I}-\xi_{i}^{I}(t)\right). \end{array}$$

We now define new variables $u_{i}^{y}(t)$ and $\theta_{i}^{y}$ as proportional to the left- and the right-hand side of these expressions,

(17) $$\begin{array}{l} u_{i}^{E}(t):=\sum_{k=1}^{M}\left\{w_{ki}^{E}\left(x_{k}(t)-{\hat{x}}_{k}^{I}(t)\right)\right\}-\beta^{E}r_{i}^{E}(t),\\ \theta_{i}^{E}:=\frac{1}{2}\left(\sum_{k=1}^{M}{\left(w_{ki}^{E}\right)}^{2}+\beta^{E}-\xi_{i}^{E}(t)\right),\\ u_{i}^{I}(t):=\sum_{k=1}^{M}\left\{w_{ki}^{I}\left({\hat{x}}_{k}^{E}(t)-{\hat{x}}_{k}^{I}(t)\right)-\beta^{I}r_{i}^{I}(t)\right\},\\ \theta_{i}^{I}:=\frac{1}{2}\left(\sum_{k=1}^{M}{\left(w_{ki}^{I}\right)}^{2}+\beta^{I}-\xi_{i}^{I}(t)\right). \end{array}$$

The variables $u_{i}^{y}(t)$ and $\theta_{i}^{y}$ are interpreted as the membrane potential and the firing threshold of neuron i of cell type y∈{E,I}.

### Dynamic equations for the membrane potentials

In this section we develop the exact dynamic equations of the membrane potentials ${\dot{u}}_{i}^{y}(t)$ for y∈{E,I} according to the efficient coding assumption. It is practical to use the vector notation and rewrite variables in Eq. (17) as

(18) $$\begin{array}{l} u_{i}^{E}(t)={\left(w_{i}^{E}\right)}^{⊤}\left(x(t)-{\hat{x}}_{I}(t)\right)-\beta^{E}r_{i}^{E}(t),\\ \left.u_{i}^{I}(t)={\left(w_{i}^{I}\right)}^{⊤}\left({\hat{x}}_{E}(t)-{\hat{x}}_{I}(t)\right)\right\}-\beta^{I}r_{i}^{I}(t),\\ \theta_{i}^{y}=\frac{1}{2}\left({\left\|w_{i}^{y}\right\|}_{2}^{2}+\beta^{y}\right)-\frac{1}{2}\xi_{i}^{y}(t), \end{array}$$

with ${\left\|w_{i}^{y}\right\|}_{2}^{2}:=\sum_{k=1}^{M}{\left(w_{ki}^{y}\right)}^{2}$ the squared length of the tuning vector of neuron i of type y. We also rewrite Eq. (8)-(9) in vector notation as

(19) $$\begin{array}{l} \dot{x}(t)=-\lambda x(t)+s(t),\\ {\dot{x}}_{E}(t)=-\lambda {\hat{x}}_{E}(t)+W_{E}f_{E}(t),\\ {\dot{\hat{x}}}_{I}(t)=-\lambda {\hat{x}}_{I}(t)+W_{I}f_{I}(t), \end{array}$$

with $\text{x}\left(t\right):={\left[x_{1}\left(t\right),\ldots ,x_{M}\left(t\right)\right]}^{⊤}$ the vector of M target signals, ${\hat{\text{x}}}_{y}\left(t\right):={\left[{\hat{x}}_{1}^{y}\left(t\right),\ldots ,{\hat{x}}_{M}^{y}\left(t\right)\right]}^{⊤}$ the vector of estimates of cell type y, and ${\text{f}}_{y}\left(t\right):={\left[f_{1}^{y}\left(t\right),\ldots ,f_{N^{y}}^{y}\left(t\right)\right]}^{⊤}$ the vector of spike trains for $N^{y}$ neurons of cell type y∈{E,I}.

In the case of E neurons, the time-derivative of the membrane potential ${\dot{u}}_{i}^{E}(t)$ in Eq. (18), is obtained as

(20) $${\dot{u}}_{i}^{E}(t)={\left(w_{i}^{E}\right)}^{⊤}\left(\dot{x}(t)-{\dot{\hat{x}}}_{I}(t)\right)-\beta^{E}{\dot{r}}_{i}^{E}(t).$$

By inserting the dynamic equations of the target signal $\dot{x}(t)$, its estimate ${\dot{\hat{x}}}_{I}(t)$ (Eq. 19) and of the single neuron readout ${\dot{r}}_{i}^{E}(t)$ (Eq. 7 in the case y=E), we get

(21) $$\begin{array}{l} {\dot{u}}_{i}^{E}(t)={\left(w_{i}^{E}\right)}^{⊤}\left[-\lambda x(t)+s(t)+\lambda {\hat{x}}_{I}(t)-W_{I}f_{I}(t)\right]-\beta^{E}\left[-\lambda_{r}^{E}r_{i}^{E}(t)+f_{i}^{E}(t)\right],\\ =-\lambda \left[{\left(w_{i}^{E}\right)}^{⊤}\left(x(t)-{\hat{x}}_{I}(t)\right)-\beta^{E}r_{i}^{E}(t)\right]+{\left(w_{i}^{E}\right)}^{⊤}s(t)-{\left(w_{i}^{E}\right)}^{⊤}W_{I}f_{I}(t)\\ -\beta^{E}\left(\lambda -\lambda_{r}^{E}\right)r_{i}^{E}(t)-\beta^{E}f_{i}^{E}(t),\\ =-\lambda u_{i}^{E}(t)+{\left(w_{i}^{E}\right)}^{⊤}s(t)-{\left(w_{i}^{E}\right)}^{⊤}W_{I}f_{I}(t)-\beta^{E}\left(\lambda -\lambda_{r}^{E}\right)r_{i}^{E}(t)-\beta^{E}f_{i}^{E}(t), \end{array}$$

where in the last line we used the definition of $u_{i}^{E}(t)$ from the Eq. (18).

In the case of I neurons, the time derivative of the membrane potential ${\dot{u}}_{i}^{I}(t)$ in Eq. (18) is

(22) $${\dot{u}}_{i}^{I}(t)={\left(w_{i}^{I}\right)}^{⊤}\left({\dot{\hat{x}}}_{E}(t)-{\dot{\hat{x}}}_{I}(t)\right)-\beta^{I}{\dot{r}}_{i}^{I}(t).$$

By inserting the dynamic equations of the population readouts of E neurons ${\dot{\hat{x}}}_{E}(t)$ and of the I neurons ${\dot{\hat{x}}}_{I}(t)$ (Eq. 19) and of the single neuron readout ${\dot{r}}_{i}^{I}(t)$ (Eq. 7 in the case y=I), we get

(23) $$\begin{array}{l} {\dot{u}}_{i}^{I}(t)={\left(w_{i}^{I}\right)}^{⊤}\left[-\lambda {\hat{x}}_{E}(t)+W_{E}f_{E}(t)+\lambda {\hat{x}}_{I}(t)-W_{I}f_{I}(t)\right]-\beta^{I}\left[-\lambda_{r}^{I}r_{i}^{I}(t)+f_{i}^{I}(t)\right],\\ =-\lambda \left[{\left(w_{i}^{I}\right)}^{⊤}\left({\hat{x}}_{E}(t)-{\hat{x}}_{I}(t)\right)-\beta^{I}r_{i}^{I}(t)\right]+{\left(w_{i}^{I}\right)}^{⊤}W_{E}f_{E}(t)-{\left(w_{i}^{I}\right)}^{⊤}W_{I}f_{I}(t)\\ -\beta^{I}\left(\lambda -\lambda_{r}^{I}\right)r_{i}^{I}(t)-\beta^{I}f_{i}^{E}I(t),\\ =-\lambda u_{i}^{I}(t)+{\left(w_{i}^{I}\right)}^{⊤}W_{E}f_{E}(t)-{\left(w_{i}^{E}\right)}^{⊤}W_{I}f_{I}(t)-\beta^{I}\left(\lambda -\lambda_{r}^{I}\right)r_{i}^{I}(t)-\beta^{I}f_{i}^{I}(t). \end{array}$$

where in the last line we used the definition of $u_{i}^{I}(t)$ from Eq. (18).

### Leaky integrate-and-fire neurons

The terms on the right-hand-side in Eqs. (21) and (23) can be interpreted as transmembrane currents. The last term in these equations, $-\beta^{y}j_{i}^{y}\left(t\right)$, y∈{E,I}, can be interpreted as a current instantaneously resetting the membrane potential upon reaching the firing threshold^28^. Indeed, when the membrane potential reaches the threshold, it triggers a spike and causes a jump of the membrane potential by an amount $-\beta^{y}$; this realizes resetting of the membrane potential which is equivalent to the resetting rule of integrate-and-fire neurons^54,56^. Thus, by taking into account the resetting mechanism and defining the time constants of population and single neuron readout $\tau =\lambda^{-1}$ and $\tau_{\tau }^{y}:={\left(\lambda_{\tau }^{y}\right)}^{-1}$, we can rewrite Eqs. (21) and (23) as a leaky integrate-and-fire neuron model,

(24) $$\begin{array}{l} {\dot{u}}_{i}^{E}(t)=-\frac{1}{\tau }u_{i}^{E}(t)+{\left(w_{i}^{E}\right)}^{⊤}s(t)-\sum_{j=1}^{N^{I}}{\left(w_{i}^{E}\right)}^{⊤}w_{j}^{I}f_{j}^{I}(t)-\beta^{E}\left(\frac{1}{\tau }-\frac{1}{\tau_{r}^{E}}\right)r_{i}^{E}(t),\\ {\dot{u}}_{i}^{I}(t)=-\frac{1}{\tau }u_{i}^{I}(t)+\sum_{j=1}^{N^{E}}{\left(w_{i}^{I}\right)}^{⊤}w_{j}^{E}f_{j}^{E}(t)-\sum_{\begin{array}{l} j=1\\ i\neq j \end{array}}^{N^{I}}{\left(w_{i}^{I}\right)}^{⊤}w_{j}^{I}f_{j}^{I}(t)-\beta^{I}\left(\frac{1}{\tau }-\frac{1}{\tau_{r}^{I}}\right)r_{i}^{I}(t),\\ \text{if}u_{i}^{y}\left(t^{-}\right)\geq \theta_{i}^{y}\to u_{i}^{y}\left(t^{+}\right)=u_{i}^{y,\text{reset}}\text{,}\\ \theta_{i}^{y}=\frac{1}{2}\left({\left\|w_{i}^{y}\right\|}_{2}^{2}+\beta^{y}-\xi_{i}^{y}(t)\right),\\ u_{i}^{E,\text{reset}}=\theta_{i}^{E}-\beta^{E},\\ u_{i}^{I,\text{reset}}=\theta_{i}^{I}-\beta^{I}-{\left\|w_{i}^{I}\right\|}_{2}^{2}. \end{array}$$

In the Eq. 24 we wrote explicitly the terms ${\left(w_{i}^{y}\right)}^{⊤}W_{x}f_{x}\left(t\right)=\sum_{j=1}^{N^{x}}{\left(w_{i}^{y}\right)}^{⊤}w_{j}^{x}f_{j}^{x}\left(t\right)$, which correspond to the synaptic projections of $N^{x}$ presynaptic neurons of type x to the postsynaptic neuron i of type y, with the quantity ${\left(w_{i}^{y}\right)}^{⊤}w_{j}^{x}$ denoting the synaptic weight. We note that, in the case of I neurons, the element with j=i describes an autapse, i.e., a projection of a neuron with itself; this term is equal to $-{\left(w_{i}^{I}\right)}^{⊤}w_{i}^{I}f_{i}^{I}(t)=-{\left\|w_{i}^{I}\right\|}_{2}^{2}f_{i}^{I}(t)$, and thus contributes to the resetting of the neuron i.

### Imposing Dale’s principle on synaptic connectivity

We now examine the synaptic terms in Eq. (24). As a first remark, we see that synaptic weights depend on tuning parameters $w_{ki}^{y}$. For the sake of generality we drew tuning parameters $w_{ki}^{y}$ from a normal distribution with vanishing mean, which yielded both positive and negative values of $w_{ki}^{y}$. This has the desirable consequence that a spike of a neuron with a positive tuning parameter in signal dimension k, $w_{ki}^{y}>0$ pulls the estimate, ${\hat{x}}_{k}^{y}\left(t\right)$, up, while a spike of a neuron with $w_{kj}^{y}<0$ pulls the estimate down, allowing population readouts to track both positive and negative fluctuations of the target signal on a fast time scale.

Another consequence of synaptic connectivity in the Eq. (24) is that the synaptic weight between a presynaptic neuron j of type x and a postsynaptic neuron i of type y is symmetric and depends on the similarity of tuning vectors of the presynaptic and the postsynaptic neuron: ${\left(w_{i}^{y}\right)}^{⊤}w_{j}^{x}=-\sum_{k=1}^{M}w_{ki}^{y}w_{ki}^{x}$. The sign of this scalar product is positive between neurons with similar tuning and negative between neurons with different tuning (and zero when the two tuning vectors are orthogonal). Thus, for a presynaptic neuron j of type x, the synaptic weights of its outgoing connections can be both positive and negative, because some of its postsynaptic neurons have similar tuning to the neuron j while others have different tuning. This is inconsistent with Dale’s principle^89^, which postulates that a particular neuron can only have one type of effect on postsynaptic neurons (excitatory or inhibitory), but never both. To impose this constraint in our model, we set synaptic weights between neurons with different tuning (i.e., ${\left(w_{i}^{y}\right)}^{⊤}w_{j}^{x}<0$ to zero. To this end, we define the rectified connectivity matrices,

(25) $$J_{ij}^{yx}={\left[\sum_{k=1}^{M}w_{ki}^{y}w_{kj}^{x}\right]}_{+},$$

with x, y∈{E,I} and ${\left[a\right]}_{+}\equiv \max\left(0,a\right)$ a rectified linear function. This manipulation is also plausible from a biological point of view, because in the cortex, the connection probability of neurons with very different (e.g. opposite) tuning is typically close to 0^51^. Since the elements of the matrix $J^{yx}$ are all non-negative, it is the sign in front of the synaptic term in the Eq. (24) that determines the sign of the synaptic current between neurons i and j. The synaptic current is excitatory if the sign is positive, and inhibitory if the sign is negative.

It is also interesting to note that rectification affects the rank of connectivity matrices. Without rectification, the product in Eq. (25) yields a connectivity matrix with rank smaller or equal to the number of input features to the network, M, similarly as in previous works^29,43,44^. Since typically the number of input features is much smaller than the number of neurons, i.e., $M\ll N^{y}$, this would give a low-rank connectivity matrix. However, rectification in Eq. (25), necessary to ensure Dale’s principle in presence of positive and negative tuning parameters, typically results in a substantial increase of the rank of the connectivity matrix.

Using the synaptic connectivity defined in Eq. (25), we rewrite the network dynamics from Eq. (24) as:

(26) $$\begin{array}{l} {\dot{u}}_{i}^{E}(t)=-\frac{1}{\tau }u_{i}^{E}(t)+\sum_{k=1}^{M}w_{ki}^{E}s_{k}(t)-\sum_{j=1}^{N^{I}}J_{ij}^{EI}f_{j}^{I}(t)-\beta^{E}\left(\frac{1}{\tau }-\frac{1}{\tau_{r}^{E}}\right)r_{i}^{E}(t),\\ {\dot{u}}_{i}^{I}(t)=-\frac{1}{\tau }u_{i}^{I}(t)+\sum_{j=1}^{N^{E}}J_{ij}^{IE}f_{j}^{E}(t)-\sum_{\begin{array}{l} j=1\\ i\neq j \end{array}}^{N^{I}}J_{ij}^{II}f_{j}^{I}(t)-\beta^{I}\left(\frac{1}{\tau }-\frac{1}{\tau_{r}^{I}}\right)r_{i}^{I}(t),\\ \text{if}u_{i}^{y}\left(t^{-}\right)\geq \theta_{i}^{y}\to u_{i}^{y}\left(t^{+}\right)=u_{i}^{y,\text{reset}}\text{,}\\ \theta_{i}^{y}=\frac{1}{2}\left({\left\|w_{i}^{y}\right\|}_{2}^{2}+\beta^{y}-\xi_{i}^{y}(t)\right),\\ u_{i}^{E,\text{reset}}=\theta_{i}^{E}-\beta^{E},\\ u_{i}^{I,\text{reset}}=\theta_{i}^{I}-\beta^{I}-{\left\|w_{i}^{I}\right\|}_{2}^{2}\text{.} \end{array}$$

These equations express the neural dynamics which minimizes the loss functions (Eq. (10)) in terms of a generalized leaky integrate-and-fire model with E and I cell types, and are consistent with Dale’s principle.

In principle, it is possible to use the same strategy as for the E-I network to enforce Dale’s principle in model with one cell type (introduced by^28^). To do so, we constrained the recurrent connectivity of the model with a single cell type from^36^ by keeping only connections between neurons with similar tuning vectors and setting other connections to 0 (see Supplementary text). This led to a network of only inhibitory neurons, a type of network model which is less relevant for the description of biological networks.

### Model with resting potential and an external current

In the model given by the Eq. (26) the resting potential is equal to zero. In order to account for biophysical values of the resting potential and to introduce an implementation of the metabolic constant that is consistent with neurobiology, we add a constant value to the dynamical equations of the membrane potentials ${\dot{u}}_{i}^{y}$, the firing thresholds $\theta_{i}^{y}$ and the reset potentials $u_{i}^{y,\text{reset}}$. This does not change the spiking dynamics of the model, as what matters to correctly infer the efficient spiking times of neurons is the distance between the membrane potential and the threshold.

Furthermore, in the same equations, the role of the metabolic constant $\beta^{y}$ as a biophysical quantity is questionable. The metabolic constant $\beta^{y}$ is an important parameter that weights the metabolic cost over the encoding error in the objective functions (Eq. 10). On the level of computational objectives, the metabolic constant naturally controls firing rates, as it allows the network to fire more or less spikes to correct for a certain encoding error. A flexible control of the firing rates is a desirable property, as gives the possibility to potentially capture different operating regimes of efficient spiking networks^36^. In the spiking model we developed thus far (Eq. 26), similarly to previous efficient spiking models^36,33^, the metabolic constant $\beta^{y}$ controls the firing threshold. In neurobiology, however, strong changes to the firing threshold that would reflect metabolic constraints of the network are not plausible. We thus searched for an implementation of the metabolic constant $\beta^{y}$ that is consistent with neurobiology.

The condition for threshold crossing of the neuron i can be written by Eq. (26) as

(27) $$u_{i}^{y}(t)+V_{\text{rest}}^{y}+\frac{1}{2}\left(c-\beta^{y}+\xi_{i}^{y}(t)\right)\geq \frac{1}{2}\left({\left\|w_{i}^{y}\right\|}_{2}^{2}+c\right)+V_{\text{rest}}^{y},$$

with c an arbitrary constant in units of millivolts. In Eq. (27) we added a constant c/2 and a resting potential $V_{\text{rest}}^{y}$ on the left- and right-hand side of the firing rule. Moreover, we shifted the noise and the dependency on the parameter β from the firing threshold to the membrane potential. Thus, we assumed that the firing threshold is independent of the metabolic constant and the noise, and we instead assumed the dependence on the metabolic constant and noise in the membrane potentials.

We now define new variables for y∈{E,I}:

(28) $$\begin{array}{l} \begin{array}{cc} V_{i}^{y}(t):\equiv u_{i}^{y}(t)+V_{\text{rest}}^{y}+\frac{1}{2}\left(c-\beta^{y}+\xi_{i}^{y}(t)\right), & V_{\text{rest}}^{y}<0, \end{array}\\ \vartheta_{i}^{y}:\equiv V_{\text{rest}}^{y}+\frac{1}{2}\left({\left\|w_{i}^{y}\right\|}_{2}^{2}+c\right), \end{array}$$

and rewrite the model in Eq. 26 in these new variables

(29) $$\begin{array}{l} \tau {\dot{V}}_{i}^{E}(t)=-\left(V_{i}^{E}(t)-V_{\text{rest}}^{E}\right)+\tau \sum_{k=1}^{M}w_{ki}^{E}s_{k}(t)-\tau \sum_{j=1}^{N^{I}}J_{ij}^{EI}f_{j}^{I}(t)-\beta^{E}\left(1-\frac{\tau }{\tau_{r}^{E}}\right)r_{i}^{E}(t)+\frac{\tau }{2}\left(c-\beta^{E}\right)+\sqrt{\frac{\tau }{2}}\sigma_{\xi }^{E}\eta_{i}^{E}(t),\\ \tau {\dot{V}}_{i}^{I}(t)=-\left(V_{i}^{I}(t)-V_{\text{rest}}^{I}\right)+\tau \sum_{j=1}^{N^{E}}J_{ij}^{IE}f_{j}^{E}(t)-\tau \sum_{\begin{array}{c} j=1\\ i\neq j \end{array}}^{N^{I}}J_{ij}^{II}-\beta^{I}\left(1-\frac{\tau }{\tau_{r}^{I}}\right)r_{i}^{I}(t)+\frac{\tau }{2}\left(c-\beta^{I}\right)+\sqrt{\frac{\tau }{2}}\sigma_{\xi }^{I}\eta_{i}^{I}(t),\\ \text{if}V_{i}^{y}\left(t^{-}\right)\geq \vartheta_{i}^{y}\to V_{i}^{y}\left(t^{+}\right)=V_{i}^{y,\text{reset}}\text{,}\\ \vartheta_{i}^{y}=V_{\text{rest}}^{y}+\frac{1}{2}\left({\left\|w_{i}^{y}\right\|}_{2}^{2}+c\right),\\ V_{i}^{E,\text{reset}}=V_{\text{rest}}^{E}-\beta^{E}+\frac{1}{2}\left(c+{\left\|w_{i}^{E}\right\|}_{2}^{2}\right),\\ V_{i}^{I,\text{reset}}=V_{\text{rest}}^{I}-\beta^{I}+\frac{1}{2}\left(c-{\left\|w_{i}^{I}\right\|}_{2}^{2}\right), \end{array}$$

where $\eta_{i}^{E}\left(t\right)$ and $\eta_{i}^{I}\left(t\right)$ are the independent Gaussian white noise processes defined in Eq. (13) above. We note that all terms on the right-hand side of Eq. (29) have the desired units of mV. The model in Eq. (29) is an efficient E-I spiking network with improved compatibility with neurobiology. We have expressed two new terms in the membrane potentials of E and I neurons, one dependent on the metabolic constant $\beta^{y}$ and one on the noise that we assumed in the condition for spiking (see Eq. 12). We will group these two terms to define an external current, a current that is well known in spiking models of neural dynamics^40^.

### Efficient generalized leaky integrate-and-fire neuron model

Finally, we rewrite the model from Eq. (29) in a compact form in terms of transmembrane currents, and discuss their biological interpretation. The efficient coding with spikes is realized by the following model for the neuron i of type y∈{E,I}:

(30a) $$\begin{array}{l} \tau {\dot{V}}_{i}^{E}(t)=-\left(V_{i}^{E}(t)-V_{\text{rest}}^{E}\right)+R_{m}\left(I_{i}^{\text{syn},E}(t)-I_{i}^{\text{ad},E}(t)+I_{i}^{\text{ext},E}(t)\right),\\ \tau {\dot{V}}_{i}^{I}(t)=-\left(V_{i}^{I}(t)-V_{\text{rest}}^{I}\right)+R_{m}\left(I_{i}^{\text{syn},I}(t)-I_{i}^{\text{ad},I}(t)+I_{i}^{\text{ext},I}(t)\right),\\ \text{if}V_{i}^{y}\left(t^{-}\right)\geq \vartheta_{i}^{y}\to V_{i}^{y}\left(t^{+}\right)=V_{i}^{y\text{,reset}}\text{,}\\ \vartheta_{i}^{y}=V_{\text{rest}}^{y}+\frac{1}{2}\left({\left\|w_{i}^{y}\right\|}_{2}^{2}+c\right),\\ V_{i}^{E,\text{reset}}=V_{\text{rest}}^{E}-\beta^{E}+\frac{1}{2}\left(c+{\left\|w_{i}^{E}\right\|}_{2}^{2}\right),\\ V_{i}^{I,\text{reset}}=V_{\text{rest}}^{I}-\beta^{I}+\frac{1}{2}\left(c-{\left\|w_{i}^{I}\right\|}_{2}^{2}\right), \end{array}$$

with $R_{m}$ the current resistance. The leak current,

(30b) $$\begin{array}{cc} I_{i}^{\text{leak},y}(t)=-\frac{C_{m}}{\tau }\left(V_{i}^{y}(t)-V_{\text{rest}}^{y}\right), & y\in \{E,I\}, \end{array}$$

with $\tau =R_{m}C_{m}$ and $C_{m}$ the capacitance of the neural membrane^54^, arose by assuming the same time constant for the target signals $x_{k}$ and estimates ${\hat{x}}_{k}^{E}$ and ${\hat{x}}_{k}^{I}$ (see Eq. 19). We see that the passive membrane time constant $\tau =\lambda^{-1}$ can be traced back to the time constant of the population read-out in Eq. (9). The synaptic currents are defined as

(30c) $$\begin{array}{l} I_{i}^{\text{syn},E}(t)=C_{m}\left(\sum_{k=1}^{M}w_{ki}^{E}s_{k}(t)-\sum_{j=1}^{N^{I}}J_{ij}^{EI}J_{ij}^{EI}\right),\\ I_{i}^{\text{syn},I}(t)=C_{m}\left(\sum_{j=1}^{N^{E}}J_{ij}^{IE}f_{j}^{E}(t)-\sum_{\begin{array}{c} j=1\\ i\neq j \end{array}}^{N^{I}}J_{ij}^{II}f_{j}^{I}(t)\right), \end{array}$$

where we note the presence of a feedforward current to E neurons,

(30d) $$\begin{array}{l} I_{i}^{\text{ff}}(t)=C_{m}\sum_{k=1}^{M}w_{ki}^{E}s_{k}(t),\\ =C_{m}{\left(w_{i}^{E}\right)}^{⊤}s(t), \end{array}$$

which consist in a linear combination of the stimulus features s(t) weighted by the readout weights $w_{i}^{E}$. The stimulus features can be traced back to the definition of the target signals in Eq. (8). This current emerges in E neurons, as a consequence of having the target signals $x_{k}\left(t\right)$ in the loss function of the E population (see Eqs. 10-11). I neurons do not receive the feedforward current because their loss function does not contain the target signal.

The current providing within-neuron feedback triggered by each spike,

(30e) $$I_{i}^{\text{ad},y}(t)=C_{m}\beta^{y}\left(\frac{1}{\tau }-\frac{1}{\tau_{r}^{y}}\right)r_{i}^{y}(t),$$

was recently recovered^37^. This current has the kinetics of the single neuron readout $r_{i}^{y}(t)$ (i.e., low-pass filtered spike train). Its sign depends on the relation between the time constant of the population readout $\tau =\lambda^{-1}$ and single neuron readout $\tau_{\tau }^{y}={\left(\lambda_{\tau }^{y}\right)}^{-1}$, because the metabolic constant $\beta^{y}$ is non-negative by definition (Eq. 10). If the single neuron readout is slower than the population readout, $\tau_{\tau }^{y}>\tau$, within-neuron feedback is negative, and can thus be interpreted as spike-triggered *adaptation*. On the contrary, if the single neuron readout is faster than the population readout, $\tau_{r}^{E}<\tau$, the within-neuron feedback is positive and can thus be interpreted as spike-triggered *facilitation*. In a special case where the time constant of the single neuron and population readout are assumed to be equal, within-neuron feedback vanishes.

Finally, we here derived the non-specific external current:

(30f) $$\begin{array}{cc} I_{i}^{\text{ext},y}(t)=C_{m}\left(\frac{c-\beta^{y}}{2}+\sigma^{y}\eta_{i}^{y}(t)\right), & \sigma^{y}=\frac{\sigma_{\xi }^{y}}{\sqrt{2\tau }} \end{array}$$

that captures the ensemble of non-specific synaptic currents received by each single neuron. The non-specific current has a homogeneous mean across all neurons of the same cell type, and a neuron-specific fluctuation. The mean of the non-specific current can be traced back to the weighting of the metabolic cost over the encoding error in model objectives (Eq. 10), while the fluctuation can be traced back to the noise intensity that we assumed in the condition for spiking (Eq. 12). The non-specific external current might arise because of synaptic inputs from other brain areas than the brain area that delivers feedforward projections to the E-I network we consider here, or it might result from synaptic activity of neurons that are part of the local network, but are not tuned to the feedforward input^69^.

We also recall the fast and slower time scales of single neuron activity:

(30g) $$\begin{array}{l} f_{i}^{y}(t)=\sum_{\alpha }\delta \left(t-t_{i}^{y,\alpha }\right),\\ \frac{dr_{i}^{y}(t)}{dt}=-\frac{1}{\tau_{r}^{y}}r_{i}^{y}(t)+f_{i}^{y}(t), \end{array}$$

and the connectivity matrices

(30h) $$\begin{array}{ccc} J_{ij}^{IE}={\left[{\left(w_{i}^{I}\right)}^{⊤}w_{j}^{E}\right]}_{+}, & J_{ij}^{II}={\left[{\left(w_{i}^{I}\right)}^{⊤}w_{j}^{I}\right]}_{+},i\neq j, & J_{ij}^{EI}={\left[{\left(w_{i}^{E}\right)}^{⊤}w_{j}^{I}\right]}_{+}. \end{array}$$

The structure of synaptic connectivity is fully determined by the similarity of tuning vectors of the presynaptic and the postsynaptic neurons ($w_{j}^{x}$ and $w_{i}^{y}$, respectively), while the distribution of synaptic connectivity weights is fully determined by the distribution of tuning parameters $w_{ki}^{y}$.

### Stimulus features

We define stimulus features as a set of k=1,…,M independent Ornstein-Uhlenbeck processes with vanishing mean, standard deviation $\sigma_{s}$ and the correlation time $\tau_{s}$,

(31) $$\tau_{s}\frac{ds_{k}(t)}{dt}=-s_{k}(t)+\sqrt{2\tau_{s}}\sigma_{s}\eta_{k}(t).$$

If not mentioned otherwise, we use the following parameters: $\sigma_{s}=2{\left(\mathrm{mV}\right)}^{1/2}$ and $\tau_{s}=10\mathrm{ms}$. Variables $\eta_{k}\left(t\right)$ are independent Gaussian white noise processes with zero mean and covariance function $\langle \eta_{k}\left(t\right)\eta_{l}\left(t^{\prime }\right)\rangle =\delta_{kl}\delta \left(t-t^{\prime }\right)$. These variables should not be confused with the Gaussian white noises $\eta_{k}^{y}\left(t\right)$ in Eq. (29).

### Parametrization of synaptic connectivity

In the efficient E-I model, synaptic weights $J_{ij}^{yx}$ are parametrized by tuning parameters $w_{ki}^{y}$ through Eq. (25). The total number of synapses in the E-I, I-I and I-E connectivity matrices (including silent synapses with zero synaptic weight) is $n_{\mathrm{syn}}=2N^{E}N^{I}+{\left(N^{I}\right)}^{2}$, while the number of tuning parameters is $n_{w}=M\left(N^{E}+N^{I}\right)$. Because the number of stimulus features M is expected to be much smaller than the number of E or I neurons, the number of tuning parameters $n_{w}$ is much smaller than the number of synapses $n_{\mathrm{syn}}$.

We can achieve a further substantial decrease in the number of free parameters by using a parametric distribution of tuning parameters $w_{ki}^{y}$. We have set the tuning parameters following a normal distribution and found that excellent performance can be achieved with random draws of tuning parameters from the normal distribution, thus without searching for a specific set of tuning parameters. This drastically decreased the number of free parameters relative to synaptic weights to only a handful of parameters that determine the distributions of tuning parameters.

Given M features, we sample tuning parameters, $w_{i}^{y}=\left[w_{1,i},\ldots ,w_{M,i}\right]$, with $i=1,\ldots ,N^{y}$, y∈{E,I}, as random points uniformly distributed on a M-dimensional sphere of radius $\sigma_{w}^{y}$. We obtain this by sampling, for each neuron, a vector of M i.i.d. standard Gaussian random variables, $\xi_{i}^{y}={\left[\xi_{1,i}^{y},\ldots ,\xi_{M,i}^{y}\right]}^{⊤}$ with $\xi_{ki}^{y}∼N\left(0,1\right)$, and normalizing the vector such as to have length equal to $\sigma_{w}^{y}$^90^,

(32) $$\begin{array}{cc} w_{i}^{y}=\sigma_{w}^{y}\frac{\xi_{i}^{y}}{{\left\|\xi_{i}^{y}\right\|}_{2}}, & y\in \{E,I\}. \end{array}$$

This ensures that the length of tuning vectors $w_{i}^{y}$ in Eq. (32) is homogeneous across neurons of the same cell type, i.e., ${\left\|{\text{w}}_{i}^{y}\right\|}_{2}=\sigma_{w}^{y}$. Parameters $\sigma_{w}^{E}$ and $\sigma_{w}^{I}$ determine the heterogeneity (spread) of tuning parameters.

By combining Eq. (25) and Eq. (32), we obtain the synaptic weights, $J_{ij}^{yx}$, as a function of the angle, $\alpha_{ij}^{xy}$, between the tuning vectors of presynaptic neurons, $w_{i}^{x}$, and postsynaptic neurons, $w_{j}^{y}$,

(33) $$J_{ij}^{yx}=\sigma_{w}^{y}\sigma_{w}^{x}{\left[\cos\alpha_{ij}^{yx}\right]}_{+}.$$

In the M=3 dimensional case, we have that the distribution of the angle between two vectors is $p\left(\alpha_{ij}^{yx}\right)=\frac{1}{2}\sin\left(\alpha_{ij}^{yx}\right)$, with $\alpha_{ij}^{yx}\in \left[0,\pi \right]$. Thus, the average strength of synaptic weights between the pre- and the postsynaptic population can be calculated as

(34) $$\begin{array}{l} \langle J_{ij}^{yx}\rangle =\frac{1}{2}\sigma_{w}^{y}\sigma_{w}^{x}{\int_{0}^{\pi }d\alpha_{ij}^{yx}\sin\left(\alpha_{ij}^{yx}\right)\left[\cos\left(\alpha_{ij}^{yx}\right)\right]}_{+}\\ =\frac{1}{4}\sigma_{w}^{y}\sigma_{w}^{x}. \end{array}$$

Thus, the upper bound for the synaptic weight between cell types x and y is simply

(35) $$max\left(J_{ij}^{yx}\right)=\sigma_{w}^{y}\sigma_{w}^{x}.$$

From the Eq. (34), we have that the mean E-I connectivity is equal to the mean I-E connectivity, $\langle J_{ij}^{EI}\rangle =\langle J_{ij}^{II}\rangle$. As we consider the ratio of the mean connectivity between E-I and I-I neurons, we find that it is given by the following:

(36) $$\frac{\langle J_{ij}^{II}\rangle }{\langle J_{ij}^{EI}\rangle }=\frac{{\left(\sigma_{w}^{I}\right)}^{2}}{\sigma_{w}^{I}\sigma_{w}^{E}}=\frac{\sigma_{w}^{I}}{\sigma_{w}^{E}}.$$

### Performance measures

#### Average encoding error and average metabolic cost

The definition of the time-dependent loss functions (Eq. 10) induces a natural choice for the performance measure: the mean squared error (MSE) between the targets and their estimators for each cell type. In the case of the E population, the time-dependent encoding error is captured by the variable $\epsilon^{E}\left(t\right)$ in the Eq. (11) and in case of I population it is captured by $\epsilon^{I}\left(t\right)$ defined in the same equation. We used the root MSE (RMSE), a standard measure for the performance of an estimator^40^. For the cell type y∈{E,I} in trial q, the RMSE is measured as

(37) $${\mathrm{RMSE}}^{y}=\sqrt{{\langle \epsilon_{q}^{y}(t)\rangle }_{t,q}},$$

with ${\langle z_{q}\left(t\right)\rangle }_{t,q}$ denoting the time- and trial-average.

Following the definition of the time-dependent metabolic cost in the loss functions (Eq. 10), we measured the average metabolic cost in a trial q for the cell type y∈{E,I} as

(38) $${\text{MC}}^{y}=\sqrt{{\langle \kappa_{q}^{y}(t)\rangle }_{t,q}},$$

with time-dependent metabolic cost $\kappa^{y}\left(t\right)$ as in model’s objectives (Eq. 11) and ${\langle z_{q}\left(t\right)\rangle }_{t,q}$ the time- and trial-average. The square root was taken to have the same scale as for the RMSE (see Eq. 37).

#### The bias of the estimator

The MSE can be decomposed into the bias and the variance of the estimator. The time-dependent bias of estimates ${\hat{x}}_{k}^{y}\left(t\right)$, y∈{E,I}, were evaluated for each time point over q=1,…,Q trials. The time-dependent bias in input dimension k=1,…,M is defined as

(39a) $$\begin{array}{l} B_{k}^{E}(t)=\frac{1}{Q}\sum_{q=1}^{Q}\left[{\hat{x}}_{k,q}^{E}(t)-x_{k}(t)\right],\\ B_{k}^{I}(t)=\frac{1}{Q}\sum_{q=1}^{Q}\left[{\hat{x}}_{k,q}^{I}(t)-{\langle {\hat{x}}_{k,q}^{E}(t)\rangle }_{q}\right], \end{array}$$

with ${\langle z_{q}\left(t\right)\rangle }_{t,q}$ the trial-averaged realization at time t. To have an average measure of the encoding bias, we averaged the bias of estimators over time and over input dimensions:

(39b) $$B^{y}=\frac{1}{TM}\sum_{k=1}^{M}\int_{0}^{T}B_{k}^{y}(t)dt.$$

The averaging over time and input dimensions is justified because $s_{k}\left(t\right)$ are independent realizations of the Ornstein-Uhlenbeck process (see Eq.31) with vanishing mean and with the same time constant, and variance across input dimensions.

#### Criterion for determining optimal model parameters

The equations of the E-I spiking network in Eqs. 30a-30h (Methods), derived from the instantaneous loss functions, give efficient coding solutions valid for any set of parameter values. However, to choose parameters values in simulated data in a principled way, we performed a numerical optimization of the performance function detailed below. Numerical optimization gave the set of optimal parameters listed in Table 1. When testing the efficient E-I model with simulations, we used the optimal parameters in Table 1 and changed only the parameters plotted in the figure axes on a figure-by-figure basis.

To estimate the optimal set of parameters $\theta =\theta^{*}$, we performed a grid search on each parameter $\theta_{i}$ while keeping all other parameters fixed as specified in Table 1. While varying the parameters, we measured a weighted sum of the time- and trial-averaged encoding error and metabolic cost. For each cell type y∈{E,I}, we computed

(40a) $$L_{\theta }^{y}=g_{L}\sqrt{{\langle \epsilon_{q}^{y}(t|\theta )\rangle }_{t,q}}+\left(1-g_{L}\right)\sqrt{{\langle \kappa_{q}^{y}(t|\theta )\rangle }_{t,q}},$$

with ${\langle z_{q}\left(t\right)\rangle }_{t,q}$ the average over time and over trials and with $\epsilon^{y}\left(t\right)$ and $\kappa^{y}\left(t\right)$ as in model’s objectives (Eq. 11).

To optimize the performance measure, we used a value of $g_{L}=0.7$. The parameter $g_{L}$ in the Eq. (40a) regulates the relative importance of the average encoding error over the average metabolic cost. Since the performance measure in Eq. (40a) is closely related to the average over time and trials of the instantaneous loss function (Eq. 10) where the parameter β regulates the relative weight of instantaneous encoding error over the metabolic cost, setting $g_{L}$ is effectively achieved by setting β.

The optimal parameter set $\theta =\theta^{*}$ reported in Table 1 is the parameter set that minimizes the sum of losses across E and I cell type

(40b) $$\theta^{*}=\underset{\theta }{\arg\min}\left(L_{\theta }^{E}+L_{\theta }^{I}\right).$$

For visualization of the behavior of the average metabolic cost (Eq. 38) and average loss (Eq. 40a) across a range of a specific parameter $\theta_{i}$, we summed these measures across the E and I cell type and normalized them across the range of tested parameters.

The exact dynamic and performance of our model depends on the realizations of random variables which describe the the tuning parameters $w_{ki}^{y}$, the Gaussian noise in the non-specific currents $\eta_{i}^{y}\left(t\right)$, and the initial conditions of the membrane potential $V_{i}^{y}\left(t=0\right)$, that were randomly drawn from a normal distribution in each simulation trial. To capture the performance of a “typical” network, we iterated the performance measures across trials with different realizations of these random variables, and averaged the performance measures across trials. We used 100 simulation trials for each parameter search.

### Functional activity measures

#### Tuning similarity

The pair-wise tuning similarity was measured as the cosine similarity^91^, defined as:

(41) $$\begin{array}{cc} \Phi_{ij}^{yx}=\cos\alpha \left(w_{i}^{y},w_{j}^{x}\right)=\frac{{\left(w_{i}^{y}\right)}^{⊤}w_{j}^{x}}{{\left\|w_{i}^{y}\right\|}_{2}{\left\|w_{j}^{x}\right\|}_{2}}, & y\in \{E,I\} \end{array},$$

with ${\left\|w_{i}^{y}\right\|}_{2}=\sqrt{\sum_{k=1}^{M}{\left(w_{ki}^{y}\right)}^{2}}$ the length of the tuning vector in Euclidean space and α the angle between the tuning vectors $w_{j}^{x}$ and $w_{i}^{y}$.

#### Cross-correlograms of spike timing

The time-dependent coordination of spike timing was measured with the cross-correlogram (CCG) of spike trains, corrected for stimulus-driven coincident spiking. The raw cross-correlogram (CCG) for neuron i of cell type y and neuron j of cell type x was measured as follows:

(42a) $$C_{ij}^{yx}(\tau )=\frac{1}{Q}\sum_{q=1}^{Q}\int_{0}^{T}f_{i,q}^{y}(t)f_{j,q}^{x}(t+\tau )dt,$$

with q=1,…,Q simulation trials with identical stimulus and T the duration of the trial. We subtracted from the raw CCG the CCG of trial-invariant activity. To evaluate the trial-invariant cross-correlogram, we first computed the peri-stimulus time histogram (PSTH) for each neuron as follows:

(42b) $$P_{i}^{y}(t)=\frac{1}{Q}\sum_{q=1}^{Q}f_{i,q}^{y}\left(t\right).$$

The trial-invariant CCG was then evaluated as the cross-correlation function of PSTHs between neurons i and j,

(42c) $$S_{ij}^{yx}(\tau )=\int_{0}^{T}P_{i}^{y}(t)P_{j}^{x}(t+\tau )dt.$$

Finally, the temporal coordination of spike timing was computed by subtracting the correction term from the raw CCG:

(42d) $$c_{ij}^{yx}(\tau )=C_{ij}^{yx}(\tau )-S_{ij}^{yx}(\tau ).$$

#### Average imbalance of synaptic inputs

We considered time and trial-averaged synaptic inputs to each E and I neuron i in trial q, evaluated as:

(43) $$\begin{array}{l} {\bar{A}}_{i,q}^{\text{net},E}=\frac{1}{TC_{m}}\int_{0}^{T}I_{i,q}^{\text{syn},E}(t)dt,\\ {\bar{A}}_{i,q}^{\text{net},I}=\frac{1}{TC_{m}}\int_{0}^{T}I_{i,q}^{\text{syn},I}(t)dt, \end{array}$$

with synaptic currents to E neurons $I_{i,q}^{\text{syn},E}\left(t\right)$ and to I neurons $I_{i,q}^{\text{syn},I}\left(t\right)$ as in Eq. (30c). Synaptic inputs were measured in units of mV. We reported trial-averages of the net synaptic inputs from the Eq. (43).

#### Instantaneous balance of synaptic inputs

We measured the instantaneous balance of synaptic inputs as the Pearson correlation of time-dependent synaptic inputs incoming to the neuron i. For those synaptic inputs that are defined as weighted delta-spikes (for which the Pearson correlation is not well defined; see Eq. 30c), we convolved spikes with a synaptic filter $F\left(t\right)=\exp\left(-\frac{t}{\tau_{syn}}\right),$,

(44) $$\begin{array}{l} A_{i,q}^{IE}(t)=\sum_{j=1}^{N^{E}}J_{ij,q}^{IE}\int_{0}^{t}f_{j,q}^{E}(t-s)F(s)ds,\\ A_{i,q}^{II}(t)=\sum_{\begin{array}{c} j=1\\ i\neq j \end{array}}^{N^{I}}J_{ij,q}^{II}\int_{0}^{t}f_{j,q}^{I}(t-s)F(s)ds,\\ A_{i,q}^{EI}(t)=\sum_{j=1}^{N^{I}}J_{ij,q}^{EI}\int_{0}^{t}f_{j,q}^{I}(t-s)F(s)ds,\\ A_{i,q}^{\text{ff}}(t)=C_{m}^{-1}I_{i,q}^{\text{ff}}(t), \end{array}$$

where we used the expression for the feedforward synaptic current from the Eq. (30d). Note that the feedforward synaptic current is already already low-pass filtered (see Eq. 31). Using synaptic inputs from the Eq. 44, we computed the Pearson correlation of synaptic inputs incoming to single E neurons, $\rho_{i,q}^{E}\left(A_{i,q}^{IE}(t),A_{i,q}^{II}(t)\right)$ for $i=1,\ldots ,N^{E}$, and to single I neurons, $\rho_{i,q}^{I}\left(A_{i,q}^{EI}(t),A_{i,q}^{\mathrm{ff}}(t)\right)$ for $i=1,\ldots ,N^{I}$. The coefficients were then averaged across trials.

#### Perturbation of connectivity

To test the robustness of the model to random perturbations of synaptic weights, we applied a random jitter to optimally efficient recurrent synaptic connectivity weights. The random jitter was proportional to the synaptic weight, ${\tilde{J}}_{ij}^{yx}=J_{ij}^{yx}\left(1+\sigma_{J}Z_{ij}^{yx}\right)$, where $\sigma_{J}$ is the strength of the perturbation and $Z_{ij}^{yx}$ are independent standard normal random variables. All three recurrent connectivity matrices (E-I, I-I and I-E) were randomly perturbed at once.

### Computer simulations

We run computer simulations with Matlab R2023b (Mathworks). The membrane equation for each neuron was integrated with Euler integration scheme with the time step of dt=0.02ms.

The simulation of the E-I network with 400 E units and 100 I units for an equivalent of 1 second of neural activity lasted approximately 1.65 seconds on a laptop.

## Supplementary Material

Supplement 1

## Figures and Tables

**Table 1.** Table of default model parameters for the efficient E-I network Parameters above the double horizontal line are the minimal set of parameters needed to simulate model equations (Eqs. 30a-30h in Methods). Parameters below the double horizontal line are biophysical parameters, derived from the same model equations and from model parameters listed above the horizontal line. Parameters $N^{E}$, M, τ and $\sigma_{w}^{E}$ were chosen for their biological plausibility and computational simplicity. Parameters $N^{I}$, $\tau_{r}^{E}$, $\tau_{r}^{I}$, σ, ratio of mean E-I to I-I synaptic connectivity and β are parameters that maximize network efficiency (see the section “Criterion for determining model parameters” in Methods). The metabolic constant β and the noise intensity σ are interpreted as global network parameters and are for this reason assumed to be the same across the E and I population, e.g., $\beta^{E}=\beta^{I}=\beta$ and $\sigma^{E}=\sigma^{I}=\sigma$ (see Eq. 3). The connection probability of $p^{xy}=0.5$ is the consequence of rectification of the connectivity (see Eq. 25 in Methods).

parameter	notation	value
number of E neurons	$N^{E}$	400
ratio of E to I neuron numbers	$N^{E}:N^{I}$	4:1
number of the input features	M	3
time constant of the population readout (E and I)	τ	10 ms
time constant of the single neuron readout	$\tau_{r}^{E}=\tau_{r}^{I}$	10 ms
noise intensity (non-specific current)	σ	5.0 (mV)^1*/*2^
heterogeneity factor of tuning parameters in E	$\sigma_{w}^{E}$	1.0 (mV)^1*/*2^
ratio of mean E-I to I-I synaptic connectivity	mean E-I : mean I-I	3:1
metabolic constant	β	14 mV

threshold constant	c/2	18 mV
distance between firing threshold and reset potential (E neurons)	$\left|\vartheta^{E}-V_{\mathrm{rest}}^{E}\right|$	19 mV
distance between firing threshold and reset potential (I neurons)	$\left|\vartheta^{E}-V_{\mathrm{rest}}^{I}\right|$	21 mV
connection probability (recurrent synapses)	$p^{IE}=p^{II}=p^{EI}$	0.5
mean E-I synaptic weight (EPSP to I at max)	$\langle J_{ij}^{IE}\rangle$	0.75 mV
mean I-E synaptic weight (IPSP to E at max)	$\langle J_{ij}^{EI}\rangle$	0.75 mV
mean I-I synaptic weight (IPSP at max)	$\langle J_{ij}^{II}\rangle$	2.25 mV

**Table 2.** Relation of time constants of single-neuron and population readout set an adaptation or a facilitation current. The population readout that evolves on a faster (slower) time scale than the single neuron readout determines a spike-triggered adaptation (facilitation) in its own cell type.

relative speed	relation of time constants	current
${\hat{x}}^{E}\mathrm{faster}thanr^{E}$ ${\hat{x}}^{E}\mathrm{slower}thanr^{E}$	$\tau <\tau_{r}^{E}$ $\tau >\tau_{r}^{E}$	adaptation in E facilitation in E

${\hat{x}}^{I}\mathrm{faster}thanr^{I}$ ${\hat{x}}^{I}\mathrm{slower}thanr^{I}$	$\tau <\tau_{r}^{I}$ $\tau >\tau_{r}^{I}$	adaptation in I facilitation in I
